# Chemical Fingerprint Imaging *In Planta* with Broadband Coherent Anti-Stokes Raman Scattering Microscopy

**DOI:** 10.1021/acs.analchem.5c01980

**Published:** 2025-07-28

**Authors:** Paul Ebersbach, Nicholas Smirnoff, Charles H. Camp, Julian Moger

**Affiliations:** † School of Physics and Astronomy, 3286 University of Exeter, Exeter EX4 4QL, U.K.; ‡ Biosciences, Faculty of Health and Life Sciences, 3286 University of Exeter, Exeter EX4 4QL, U.K.; § Biosystems and Biomaterials Division, 10833 National Institute of Standards and Technology, 100 Bureau Dr., Gaithersburg, Maryland 20899, United States

## Abstract

Plants are inherently complex systems dynamically interacting at different size scale levels. Spontaneous Raman microscopy links the molecular with the cellular structural level; however, as Raman scattering is a low-probability phenomenon, pixel dwell times for biological applications are not compatible with high-resolution imaging. Due to absorption and autofluorescence interferences, Raman methods are often restricted to pigment-poor regions in plant samples. Here, we apply broadband coherent anti-Stokes Raman scattering (BCARS) microscopy—a nonlinear optical counterpart of spontaneous Raman microscopy—for the first time on plant samples. We show that it generates Raman-like vibrational signals but with much faster acquisition times (10 ms/spectrum), facilitating large-area imaging in high resolution. Using a new optimized unmixing procedure in conjunction with existing, robust preprocessing methods, we can extract the chemical and spatially rich information from leaf cross sections from the upper cuticle to the chlorophyll fluorescence-dominated palisade and spongy mesophyll region. The method selectively extracts chemical components from the cuticle (waxes), cell walls (pectin, cellulose), and mesophyll (chlorophyll, carotenoids, lipoproteins, starch) and depicts the accumulation of calcium oxalate crystals, flavonols, and anthocyanins in vacuoles. Photosystem-specific spectral changes of chlorophyll and carotenoid signals in intact and degraded leaves reveal a chloroplast adaptation to the light absorption gradient in a leaf. The signal-intense bands give rise to further enhancement mechanisms through electronic (pre)­resonance (chlorophyll, anthocyanin), strongly coherently amplified supramolecular ensembles with copigments (anthocyanin), and π-electron–phonon coupling (carotenoids). OH-stretching signals reveal that calcium oxalate crystals have a mixed hydration state. The results outline that the system view imaging capabilities of BCARS microscopy make it a valuable tool in plant and agrochemical research.

## Graphical abstract

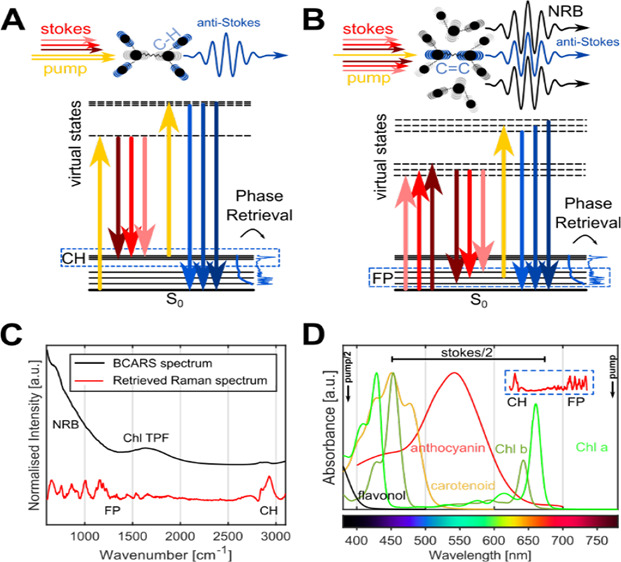

## Introduction

As well as providing food, plants synthesize specialized compounds with a wide range of uses. Therefore, understanding plant metabolism is central to improving crop productivity and engineering the increased production of useful products. An important requirement is to know the distribution of compounds between cell types and their subcellular localization in organelles, cytoplasm, and vacuoles. Analytical techniques, such as mass spectrometry imaging and “single cell” sampling, are limited in spatial resolution, cannot easily achieve subcellular resolution, and require extensive sample processing.^1^ An alternative approach is to use genetically encoded sensors (based on GFP or other fluorescent proteins) whose expression can be directed to specific subcellular locations. These sensors change their fluorescence spectra when interacting with target compounds and have been designed to detect specific metabolites (e.g., glucose), pyridine nucleotide and glutathione redox state, ATP, hormones, and signaling molecules.^2–4^ The use of biosensors is limited by the impracticality of detecting multiple compounds and plant species that are amenable to genetic transformation and may compete with endogenous fluorescence. Such autofluorescence can be a useful tool for label-free imaging. Phenolic-containing polymers such as lignin and ferulates enable cell wall imaging.^5^ The pulse amplitude modulation (PAM) fluorimetry of chlorophyll a in the photosystem II reaction center provides information on photosynthetic efficiency, electron transport rates, and energy dissipation and can reach single chloroplast resolution.^6,7^

Optical techniques based on vibrational spectroscopy, such as infrared (IR) and Raman spectroscopy, rely on light–matter interactions mediated by molecular vibrations. Often termed a chemical fingerprint (FP), these provide dense spectra of narrow lines corresponding to the unique vibrational modes of molecules. They are intricate, particularly in complex, mixed biological samples, and in conjunction with chemometric methods, enable dense multiplexed analysis of a wide range of chemical species simultaneously. However, in terms of high-resolution biological imaging, IR has limited value due to water absorption and the intrinsically low spatial resolution resulting from IR excitation. Although Raman scattering can probe vibrational frequencies with biocompatible excitation wavelengths, it is an extremely weak effect, with typical photon conversion efficiencies in biological materials being approximately 10^6^ times weaker than fluorescence. FP spectra of sufficient quality in biological samples are only feasible with collection times ranging from 0.1 to 30 s per pixel, precluding the use of Raman spectroscopy in high-resolution bioimaging and its many potential powerful uses in biological research. In plant tissues, the lack of sensitivity is compounded by interference from autofluorescent pigments, which overlap and overwhelm the Raman emission, significantly limiting its application for imaging in plant biology.

Coherent Raman methods use pulsed laser sources to coherently drive molecular vibrations, leading to more intense spectral signals with similar average power sources.^8^ Of these, narrowband coherent anti-Stokes Raman scattering (CARS) and stimulated Raman scattering (SRS) have emerged as widely adopted techniques for high-speed biological microscopy.^9^ CARS has been applied to visualize ß-carotene in plant cells^10^ and cannabinoids and terpenes in single trichomes.^11^ SRS has been demonstrated to overcome the strong autofluorescence in plant tissues^12^ and has been applied to visualization of epicuticular waxes,^13,14^ biomass conversions,^15^ characterization of cell walls,^16,12^ and agrochemical depositions.^12^ These methods use narrowband pump and Stokes beams, with their frequency difference matched to a wavenumber of interest, to focus the excitation energy onto a single vibrational mode and generate a strong Raman signal. While this can enable, via video rate, selective contrast of molecules with unique Raman lines, it does not make use of the wealth of chemical information contained within the vibrational spectrum. Broadband excitation schemes, such as spectral-focusing SRS,^17^ and dual- and multichannel SRS,^18,19^ have been demonstrated to provide hyperspectral imaging over the 300 cm^–1^ range covering the high wavenumber CH region. However, extending the bandwidth to cover the FP region while maintaining the spectral energy density requires excitation powers that are incompatible with biological samples. This is further compounded by weaker signals in the FP regions.

Exploiting the potential of Raman spectroscopy for chemical imaging requires probing broad swaths of the biologically relevant spectral window. To this end, broadband CARS (BCARS) uses a broadband Stokes source that interacts with a narrowband probe to generate a broad spectrum of inelastic scattering from the probe source. Traditional implementations of BCARS (also known as multiplex CARS [MCARS] in this context) used photons from the narrowband source for pump and probe photons—a paradigm known as two-color/interpulse stimulation (Figure 1A). Despite generating broad spectra, results with such systems have historically suffered from poor detection limits due to the low spectral energy density of broadband sources at average powers below the damage threshold of biological samples. A breakthrough in BCARS imaging was made by Cicerone and co-workers in 2014,^20^ who developed a new BCARS configuration that enables the collection of high-quality Raman spectra in milliseconds and with high signal-to-noise ratios, especially in the information-rich Raman FP region (∼500 to 2000 cm^–1^). In the new system within the FP region, the broadband source provides both the pump and Stokes photons, while the narrowband source solely provides the probe photon—and arrangement known as three-color/intrapulse stimulation (Figure 1B), which dramatically increases the spectral energy density of the stimulation profile disproportionately at the smallest wavenumbers for a given average power (it also has reduced spectral noise characteristics for the broadband source). In addition, by a careful selection of laser center wavelengths, this system also takes advantage of two-color stimulation at higher frequencies; thus, both stimulation pathways together provide Raman spectra with bandwidths over 3500 cm^–1^.

**Figure 1:**
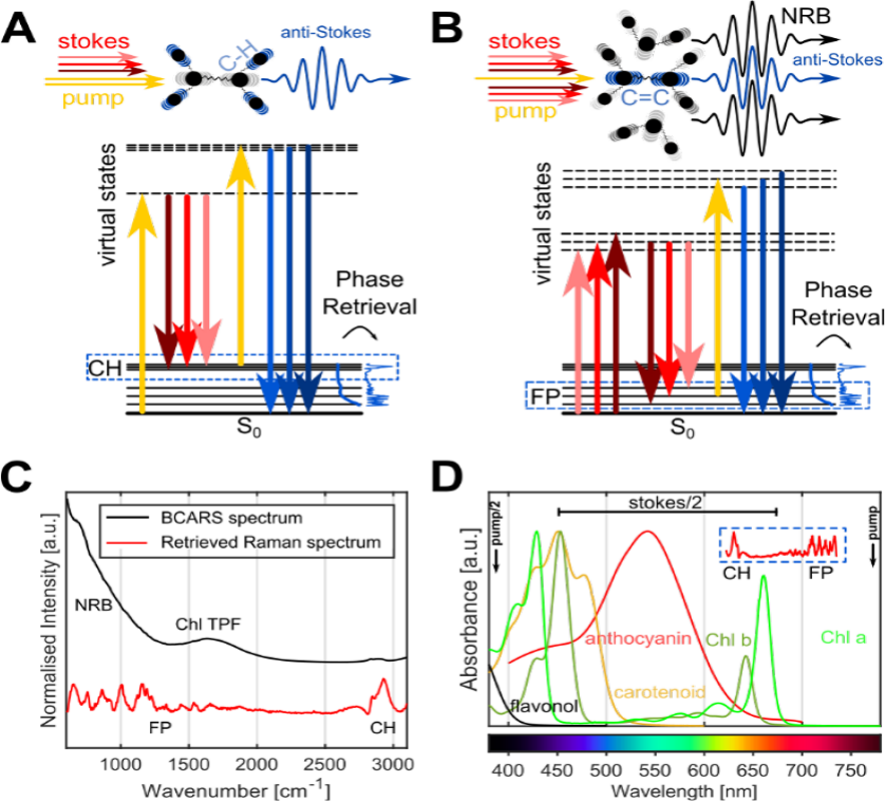
Illustration of the BCARS processes and data processing. (A) Scheme and energy diagram with two-color excitation. (B) Scheme and energy diagram with three-color excitation. (C) Mean BCARS spectrum and corresponding retrieved Raman spectrum taken from a plant leaf cross-section spectral image. (D) Absorbance spectra of plant pigments and a flavonol. The inset shows the spectral range of the BCARS light. Chlorophyll a and b in diethyl ether; carotenoid: beta-carotene in hexane; flavonol: rutin trihydrate in methanol; anthocyanin: cyanidin-3-glucoside in water at pH 1.^23,24^ CH, CH-stretching region; FP, fingerprint region; NRB, nonresonant background; TPF, two-photon fluorescence.

A complication of BCARS is that the spectra are distorted due to the cogeneration of a so-called nonresonant background (NRB) resulting from the electronic (nonvibrational) interaction of the incident light and the sample. Beneficially, the NRB acts as a strong, phase-locked homodyne amplifier, bringing vibrational signals above the noise floor at the expense of the spectra being distorted from frequency-dependent constructive and destructive interference. This interfering relationship, though, between the vibrational components and the NRB is fixed such that “phase retrieval” methods may be used to remove such distortions in postprocessing, extracting Raman-like spectra. These methods, however, assume that the electronic response is far from resonance and that there is no electronic-vibrational coupling. However, for plant imaging applications, this assumption might not hold as the energy of the generated anti-Stokes photons and the half energy of the pump and Stokes photons are close to electronic transitions of the common plant pigments giving rise to phenomena like (two-photon) absorption and fluorescence, and (pre)­resonance enhancement effects, which might also modulate the NRB (Figure 1D). Indeed, BCARS spectra clearly show a broad peak in the FP region, which can be assigned to two-photon fluorescence from chlorophyll (Figures 1C and S1–S2).

Here, we demonstrate that despite strong autofluorescence and absorption, BCARS can retrieve full Raman information and map the relative abundance of chemical species *in planta* (Figure 1C) using phase retrieval and a unique hyperspectral unmixing method presented later. We herein demonstrate the strength of this method even in chlorophyll-rich regions where absorption and autofluorescence have previously prohibited imaging with all other Raman-based methods.^21,22^ This advance provides a new tool to image variation in the accumulation of specialized metabolites between cell types and specific subcellular compartments.

## Experimental Section

### Sample Collection and Preparation

Leaves of the evergreen “Emerald Gaiety” were freshly collected on the campus of the university, grown under sun-exposed, south-oriented natural light conditions in December 2020 (Exeter, UK, 50°44′11.1”N 3°32′15.5”W). These leaves show pronounced variegation, facilitating imaging of different colored zones in a leaf. We focused the study on green- and red-green-colored regions found in some of the leaves. Leaf cross sections (thickness 30 μm) were obtained via a hand microtome cut with razor blades and immediately placed in water. For imaging, the cuts were placed on a drop of water on a coverslip. A second coverslip was tightly pressed on the first one and sealed with a self-adhesive spacer.

### BCARS Microscope

The microscope is based on the configuration described by Camp et al.^20^ The pump beam (770 nm; 3.4 ps) and broadband Stokes beam (900–1350 nm; ∼16 fs) are generated from two coseeded fiber lasers (Toptica Photonics, FemtoFiber pro), with powers measured at the sample plane of 8.8 and 9.6 mW, respectively. No photodamage was observed while imaging with these powers in both pigmented and nonpigmented regions. The spectral resolution of this configuration, determined by the bandwidth of the (770 nm) probe, is ∼10 cm^–1^. The two beams are temporally superimposed via a motorized delay line (ThorLabs, PLS-X), coupled into an inverted microscope (Olympus, IX71), and focused on the sample using a water-immersion objective lens (Olympus, UPlanSApo IR, NA = 1.2, ×60; controlled via ThorLabs, PLS-X). An xyz-piezo stage (Physik Instrumente, P-545 and E-727 controller) enables 200 μm movements of the mounted sample in each direction with submicrometer precision. A second stepper motor stage below the piezo stage is used for large-area, coarse resolution movements (Prior Scientific, H118 and ProScan III controller). The emitted light is collected and collimated with a ×60 objective lens (Olympus, LUCPlanFL N, NA = 0.7) and short-pass filtered (Semrock, Brightline 770SP; Chroma, HHQ765SP). The transmitted signal was focused onto the slit of a spectrograph (IsoPlane 160, Princeton Instruments) equipped with a CCD camera (PIXIS 400BR_eXcelon) for spectral recording. The camera acquisition is synchronized with the x-movement of the piezo stage via pixel-wise trigger signals generated from the piezo controller, resulting in a pixel dwell time of 10 ms (including readout time). The interaction of the motors (focus of objectives and pump delay), movement of the two stages, and the spectral acquisition are controlled via a graphical user interface programmed in MATLAB R2020a (Mathworks). BCARS hyperspectral imaging of “Emerald Gaiety” was performed at areas of 195 × 200 μm, focused at a depth corresponding to the center of the 30 μm-thick section, with a 0.5 μm resolution, resulting in *x*-*y*-λ stacks of size 390 × 400 × 1340. The total acquisition time, including stage *x*-direction return time between line-scans, is <30 min.

### Data Processing

The hyperspectral data are processed in a graphical user interface programmed in MATLAB R2020a. First cosmic rays are identified via a local maxima search in the spectral-spatial space and replaced via inward interpolation along the spatial dimension. Further preprocessing steps are based on the approach described in further detail by Camp et al.:^25^ first, the data are variance stabilized using an Anscombe transform, which converts signals with mixed Poisson noise (shot noise) and additive white Gaussian noise (AWGN) into a signal with just AWGN. Singular value decomposition (SVD) is used to decompose the signal and has been demonstrated to more optimally divide noise and signal after such an Anscombe transformation.^16,26^ Singular values, which exceed a specific ratio of the high-frequency/low-frequency components in the (2D) Fourier transformed spectral or spatial space, are assumed as noise components and removed after manual inspection. An exact, unbiased inverse Anscombe transformation is applied to return the signal to its original scale.^27^ Phase retrieval is performed using a Hilbert transform with cover glass spectra as the NRB reference material.^26^ Owing to differences in the actual pixel-by-pixel NRB and that of coverslip glass, retrieved phase errors and multiplicative amplitude errors result but can be removed by the phase- and scale-error correction method described by Camp et al.^25^ The imaginary part of the spectra is then concatenated into FP- and CH-stretching regions and vector normalized prior to the unmixing to only account for spectral shape variations.

Our linear unmixing approach of the Raman-like hyperspectral data set is separated into an endmember extraction step (EEA) followed by the actual unmixing step (Figure 2). For the endmember extraction, we used the maximal distance algorithm (MaxD),^28^ which is similar to the popular vertex component algorithm (VCA),^29^ which identifies the endmember spectra as the extreme pixels located at the edges of the data cloud in the spectral space via subsequent orthogonal projections.^30^ In contrast, MaxD shows less randomness in the endmember extraction,^30^ which can also induce slight variations in the abundance maps retrieved via the unmixing (Figures S4 and S5). Additionally, the MaxD searches for the most extreme pure pixels and is thus more sensitive to outliers but possibly also allows extraction of more subtle spectral features compared to the VCA. To effectively remove outliers and simplify the endmember search in our high pixel number images, we applied the endmember extraction algorithm (EEA) on spatially 2D median-filtered data sets, highly reducing the complexity of the data set. This quite drastic processing step assumes that the pure pixels lie in homogeneous pixel regions and thus show very similar spectra in their neighborhood. This assumption is only valid if the 2D median filter window size is correctly chosen, which we inspected via a similarity analysis of the single neighborhood pixel spectra after performing the EEA. We identified a 5 × 5 size neighborhood—each endmember spectrum is a 2D median spectrum of 25 spectra—as the right size showing no mixing in our data sets (Figure S6). Furthermore, we tested unmixing models based on 3 × 3, 5 × 5, and 7 × 7 2D median-filtered endmember spectra, which confirmed that a neighborhood of 5 × 5 provided an optimal trade-off between spectral and spatial specificity (Figure S7).

**Figure 2:**
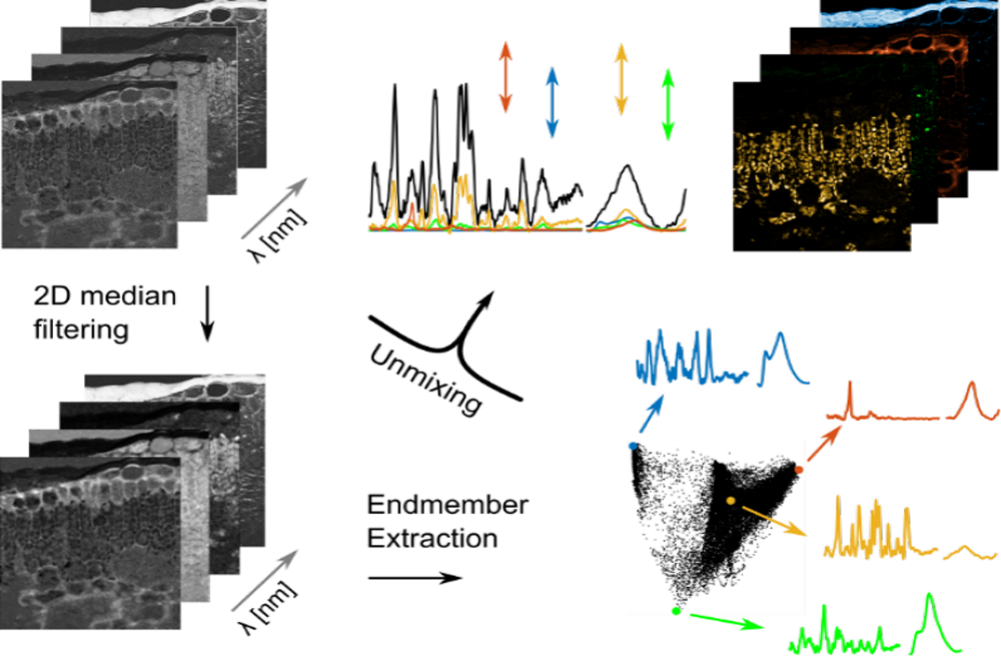
Schematic of the unmixing procedure. Unmixing is performed on the unfiltered data set, and the endmember spectra are retrieved from the 2D median-filtered data set.

In the actual unmixing step, the averaged pure pixel spectra are fitted toward the original data set, which maintains the original contrast and prevents the creation of blurry effects at the edge regions, which are often visible after median filtering. We then apply a fast combinatorial nonnegative-least-squares algorithm.^31^

## Results

We applied our unmixing procedure to a green-colored part of a leaf of . The number of endmembers was varied between 10 and 20. Based on visual inspection, 14 endmembers were found to most suitably depict the spectral variations of the leaf. The spectral range was restricted toward the FP- and CH-stretching regions from 600 to 1800 cm^–1^ and 2800 to 3100 cm^–1^, respectively. Figure 3 shows the endmember spectra and corresponding abundance maps sorted in a specific arrangement. Cuticles, cell walls, and vascular bundles are mainly depicted by EM1–4 (Figure 1C). To illustrate the necessity of the unmixing and phase retrieval processes, the average raw spectra corresponding to each endmember are shown in the Supporting Information (Figure S8). Epidermal cell and mesophyll spectral features are reflected by EM5–6 and EM7–10, respectively (Figure 1D,E). EM11–14 reveals unique Raman bands, which can be attributed to calcium oxalate crystals (Figure 1F). In the following sections, the four groups are separately discussed in detail. Further information is obtained from the unmixing results from the red-green part of a leaf (Figure S9). Peak assignments with spontaneous Raman bands from the literature are shown in Table S1.

**Figure 3:**
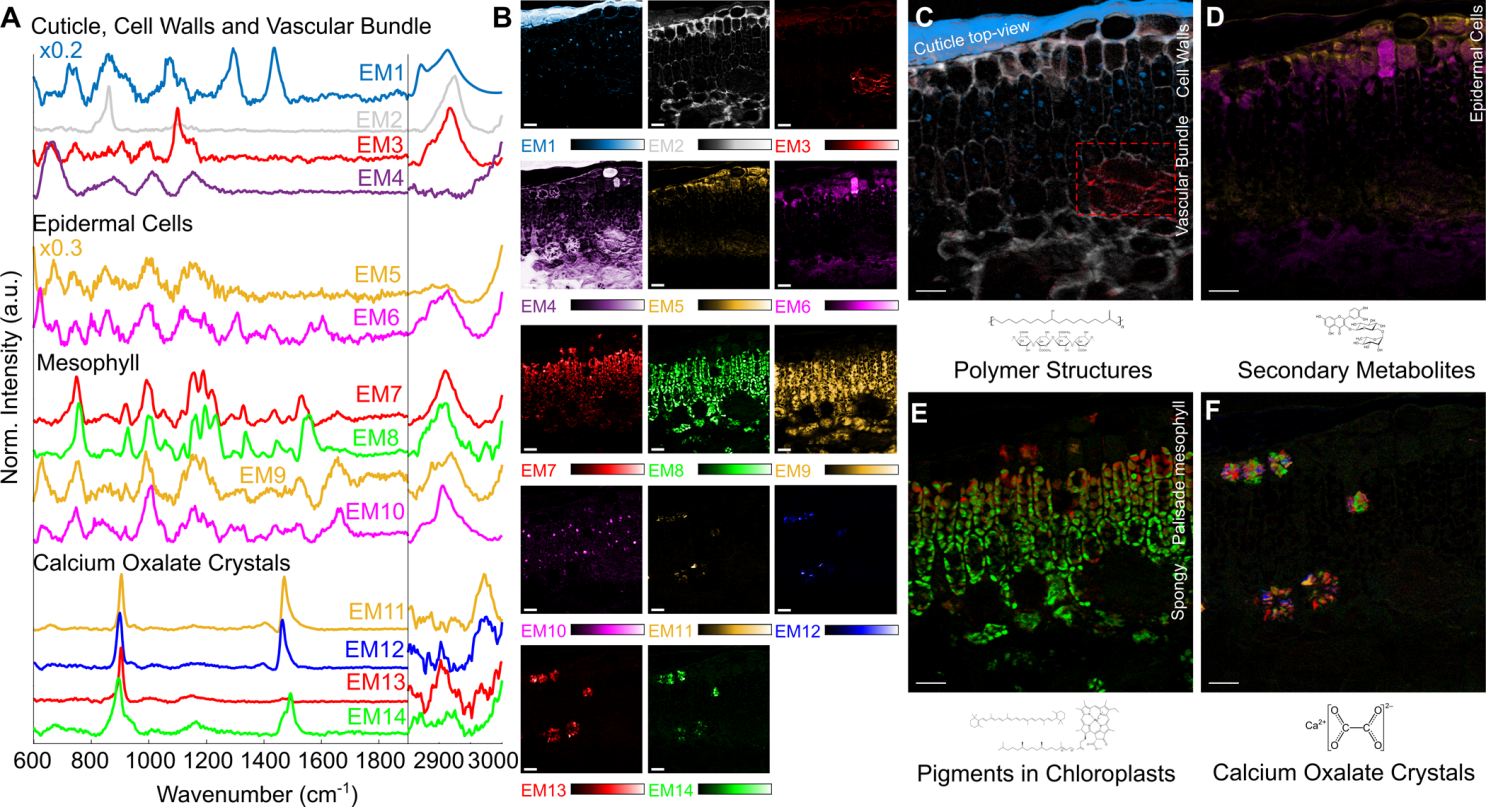
Unmixing results of a plant leaf cross section of ; Scale bars: 20 μm. (A) Normalized endmember spectra. Note, for better visualization, the FP spectra in EM1 and EM5 have been scaled up by factors of 5 and 3, respectively. (B) Corresponding abundance maps. (C) Overlay image of EM1, EM2, and EM3, depicting polymer structures in the cuticle, cell walls, and vascular bundle, respectively. The apparently thick cuticle is a sample-squeezing artifact showing it from top view. (D) Accumulation of secondary metabolites inside the epidermal cell vacuoles depicted by EM6. (E) Overlay image of EM7 and EM8 depicting pigments inside the palisade and spongy mesophyll. (F) Overlay image of EM11–14 depicting calcium oxalate crystal spectral features.

We also applied our unmixing procedure on raw BCARS data prior to the phase retrieval to obtain two-photon fluorescence (TPF) abundance maps (Figure S10).

### Cuticles, Cell Walls, and Vascular Bundles

The EM1 abundance map mainly depicts the cuticle and, with less abundance, the cell walls and nuclei of the palisade cells. It is spectrally characterized by a signal intensity dominating lipid-like CH-stretching and further lipid-like FP signals (1293 and 1434 cm^–1^) and pectin signals (860, 1073, and 1110 cm^–1^), which are in accordance with bands found in a tomato cuticle via spontaneous Raman spectroscopy.^32^ An additional band at 723 cm^‑1^ can be assigned to δ­(CH_2_) rocking of cuticular waxes.^33^ Less-intense signals reveal the presence of phenolic compounds (1601 and 1639 cm^–1^).^32^ EM2 uniquely highlights the cell wall arrangement via the pronounced Raman band at 860 cm^–1^, indicating the α-Glycosidic bonds in pectin and further low-intense signals of asymmetric and symmetric (C–O–C) stretching in cellulose (1099 and 1154 cm^–1^). The signal intensity of the asymmetric stretching at 1099 cm^–1^ is dependent on the cellulose microfibril orientation in the cell walls when exciting with polarized light.^34^ In EM3, this band is different from EM2 pronounced and only highly abundant in a specific structure, which can be assigned to the vascular bundle. In the cell walls, this orientation-dependent signal indicated by EM3 is less abundant. It is known that the four-wave mixing excitation in CARS is polarization-dependent for molecules with ordered orientation.^35^ This polarization dependency favors the excitation of a specific orientation arrangement of the cellulose in the vascular bundle.^36^ The cell wall arrangement depicted by EM3 separates the vascular bundle into an upper (adaxial) part and a bottom (abaxial) part, the xylem and phloem, respectively. EM4 is highly abundant at the outside of the leaf and is characterized by background FP signals originating from the glass cover slide (Figure S11). Increased abundances inside the leaf display transparent regions dominated by the background signals. These are some cells of the upper epidermis, the surrounding of the calcium oxalate crystals, the vascular bundle (xylem more than the phloem), and the spongy mesophyll.

### Epidermal Cell Content

The EM5 and EM6 abundance maps together describe the vacuole content of epidermal cells. EM5 shows some FP features of EM6 with lower intensity, especially less-intense CH-stretching signals. It possibly represents EM6 or a similar component in a low-concentration environment. The more intense FP signals in EM6 reveal the identity of the component: the characteristic stretching vibrations of aromatic C=C groups (1564 and 1604 cm^–1^) and further dihydroxyphenyl and mannopyranosyl ring bands hint at the accumulation of a flavonol similar to rutin, a glycoside of quercetin commonly found in plants.^37–39^ The NMR analysis of isolated compounds of identified different quercetin glycosides, proving the accumulation of one or more of these compounds inside the epidermal cell vacuoles.^40^

### Chloroplast Spectral FP Gradient in a Green Leaf

The palisade and spongy mesophyll contrasts are mostly generated by EM7–10. EM7, 8, and 9 together depict pigment spectral features inside the chloroplasts. EM10 depicts the nuclei of the palisade cells partly colocalizing with EM1. The strong phenylalanine band at 1010 cm^–1^ and amide I band at 1670 cm^–1^ reveal the presence of proteins,^41^ while EM1 represents the lipid fraction inside the nuclei.

Figure 4A–C compares the spectral characteristics and abundance maps of EM7 and 8, giving insights into the chloroplast pigment distribution. The spectra in Figure 4A reveal bands from chlorophyll and carotenoid molecules, which are probably enhanced via electronic (pre)­resonance and π-electron–phonon coupling, respectively (Figure S3). The carotenoid bands are separated into three main frequency regions: In-phase C=C stretching (ν_1_ region: 1530–1542 cm^–1^) and C–C stretching (ν_2_ region: 1150–1250 cm^–1^) of the polyene chain and in-plane (CH3) rocking coupled with C–C bonds of the polyene chain (ν_3_ region: 990–1010 cm^–1^).^42^ The most characteristic chlorophyll bands are located at ∼750, ∼1290, and ∼1330 cm^–1^.^43^ The pronounced band at ∼750 cm^–1^ is attributed to in-plane deformation of the pyrrole ring.^44^ A shoulder peak of the ν_1_ band at ∼1550 cm^–1^ suggests a 5-coordination in the chlorophyll a molecule.^45^

**Figure 4:**
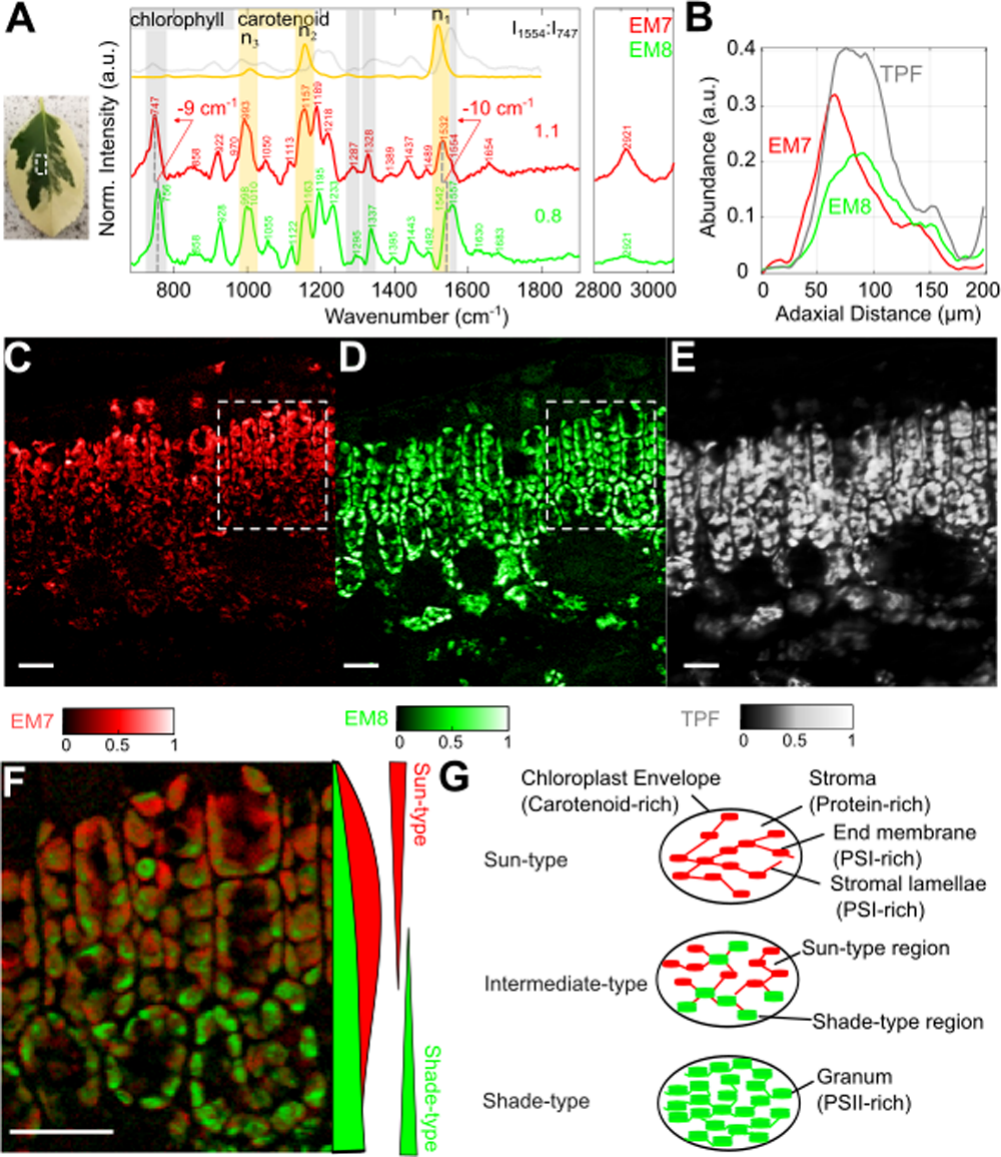
Intraleaf mesophyll gradients in a cross section of a green region of . (A) Endmember spectra; (B) mean abundance profiles along the distance from the adaxial surface (3-polynom Savitzky-Golay filtered with frame length 61); (C–E) abundance maps of EM7, EM8, and TPF; (F) zoomed overlay image of EM7 and EM8 and mean abundance along the adaxial–abaxial axis (3-polynom Savitzky-Golay filtered with frame length 91); (G) ultrastructure in different chloroplast types. Reference spectra: β-Carotene, CAS: 7235–40–7 (orange), chlorophyll a from spinach CAS: 479–61–8 (gray). Scale bars: 20 μm.

EM7 shows the highest abundances in the upper chloroplasts of the palisade cells and in some epidermal cells (Figure 4C). The abundances gradually decrease along the adaxial-abaxial axis of the leaf. EM8 shows a more uniform distribution over all chloroplasts of the palisade and mesophyll cells. In the upper palisade cells, EM7 is more spread toward the edges of the chloroplast possibly also depicting pigments of the outer chloroplast regions, e.g., the chloroplast envelope, while EM8 shows only high abundances in the center, for example, thylakoids (zoomed inset images in Figure 3C,D). The average maximum abundance of EM7 is shifted toward the adaxial side of the leaf in comparison to EM8 (Figure 4B).

Relative signal intensity differences are observed for the chlorophyll and carotenoid bands, which are here quantified via the ratio of the pronounced carotenoid signal intensity at 1157 cm^–1^ and the chlorophyll signal intensity at 747 cm^–1^ (*I*_1157_:*I*_747,_ Figure 4A). EM7 shows a relatively higher carotenoid signal intensity with *I*_1157_:*I*_747_ = 1.1 in comparison to EM8 with *I*_1157_:*I*_747_ = 0.8. The chlorophyll a specific shoulder peak at 1554 cm^–1^ decreases relatively to the neighboring ν_1_ band. The low-intensity signals in EM8 at 1630 and 1683 cm^–1^, which can be attributed to formyl stretching in chlorophyll b and keto stretching in chlorophyll a,^46^ completely disappear in EM7, which shows a broad peak at 1654 cm^–1^ and is probably attributed to the amide I band.^43^ This band together with an increased protein-like CH-stretching hints at an increased protein content. The entire EM7 spectrum is red-shifted with respect to the spectrum of EM8, which is most obvious in the ν_1_ band with a shift of 10 cm^–1^.

A colocalization analysis of the abundance maps of EM7 and EM8 with TPF gives further insights (Figure 4C–E). Regions of EM7 abundances >0.5 colocalize with regions of quenched fluorescence emission. The scatter plot in Figure 4C clarifies that the maximum achievable TPF in a pixel gradually decreases with increasing EM7 abundance >0.5 in that pixel and saturates at 0.5. Such a limitation is not observable for pixels with a high abundance of EM8, which can also show a high TPF (Figure 4D, scatter plot). Thus, EM7 might be a spectral FP for a fluorescence quenching state.

At first glance, the smooth, gradual decrease of the EM7 abundances seems to be an optical artifact often observed in thin sample imaging, generated by a slightly tilted sample plane or changing sample thickness along the adaxial–abaxial axis. However, this adaxial-abaxial gradient corresponds to depth-dependent light exposure originating from an adaptation mechanism of the chloroplasts toward their local light environment.^47^ Due to the gradually increasing light absorption through the leaf, the upper chloroplasts experience higher light fluxes and differences in the light spectrum in comparison to the lower chloroplasts. The gradient of light causes a long-term acclimatization (hours to days) of the leaf as a function of leaf depth, increasing the photosynthetic capacity toward the adaxial side.^48^ Short-term excess light exposure (seconds to minutes) may further cause a reversible gradient in photoprotection or an irreversible gradient in photoinhibition–photoinactivation of photosystem II (PSII).^49,50^ All these photomechanisms underlie complex, highly dynamic structural changes happening on different time and length scales (single pigments to whole thylakoid network structure) and can only be investigated under well-defined light conditions.^51^

In this study, we do not aim to go in detail into photosystem investigations growing the plant under natural, highly dynamic light conditions and neglecting short-term light responses during sample preparation and measurement. Still, the observed characteristics of EM7 as opposed to EM8 (as a kind of internal reference) may allow a plausible explanation for the gradient phenomenon.

The blurry distribution of abundances of EM7 might reflect less densely packed thylakoid structures, which are typically found in adaxial, high-light-grown or sun-type chloroplasts.^48^ These chloroplasts with increased photosynthetic capacity have fewer and shorter grana stacks and thus possibly show higher spectrochemical contributions of the stroma-exposed membranes, stroma, and chloroplast envelope, reflected here by EM7. An increased carotenoid-to-chlorophyll ratio observed here via relative band ratios is typical for sun-type chloroplasts and is primarily caused by higher levels of xanthophyll cycle carotenoids^49,52,53^ but may also originate from an increased contribution of the chloroplast envelope membrane containing carotenoids but no chlorophyll.^54^ The increased protein bands with respect to the chlorophyll bands in EM7 may reflect an upregulation of the protein rubisco inside the stroma,^47,55^ relatively to the light-harvesting machinery reflected by the chlorophyll bands. This and the observed fluorescence quenching at high EM7 abundances might hint at an increased photosynthetic capacity of adaxial chloroplasts. However, the reduced fluorescence might also originate from the increased contribution of the stroma membrane optical properties, showing a reduced fluorescence emission.^56^

On the other hand, the more contrast-rich image of EM8 might depict spectral contributions originating more from a densely packed thylakoid network, which is found in abaxial, low-light-grown or shade-type chloroplasts—these have more and taller grana and thus a relatively higher amount of PSII-rich grana^57^ (Figure 4O). Thus, shade-type chloroplasts typically show a decreased PSI:PSII ratio, adapting their photosynthetic machinery to light that is enriched in far-red wavelength, possibly reflected here by an overall blue-shifted spectrum and increased chlorophyll Raman band and fluorescence emission.

Schumann et al.^49^ observed shared sun- and shade-type thylakoid structural as well as molecular features of chloroplasts grown under natural light conditions and attributed this to a better adaptation toward highly fluctuating light conditions. In accordance with that, we speculate that the locally increased EM 7 abundances reflect sun-type regions, while the relatively increased EM 8 abundances reflect shade-type regions. As the relative amount of the two regions gradually changes, we speculate that the intraleaf adaptation mechanism is regulated by the relative amount of these two types and gradually changes from almost only sun-type regions in adaxial chloroplasts (here in epidermal chloroplasts) toward almost only shade-type regions in abaxial chloroplasts.

### Chloroplast Spectral FP Gradient in a Red-Green-Colored Leaf

We further applied our unmixing on a hyperspectral image of a red-green leaf with a potentially degraded photosystem (Figure S9). Figure 5C,D compares the endmembers EM23–24, depicting chloroplast pigment spectral features. Similar to the green leaf, we see here a pigment spectral feature gradient, which can be attributed to different light fluxes in the upper and lower leaf; however, with much higher impact, EM23 is highly abundant at the adaxial side, while EM24 is more abundant at the abaxial side. The spectrum of EM23 is almost similar to a pure carotenoid spectrum^42^ and dominated by the ν_1_–ν_3_ bands at 1530, 1157, and 1006 cm^–1^, respectively, implying a strong decrease of chlorophyll in the upper leaf, which is also assessed via the carotenoid:chlorophyll band ratio of *I*_1157_:*I*_747_ = 4.6. Low chlorophyll concentration is further suggested by the strong TPF decrease toward the upper leaf (Figure 3J,M). The spectrum of EM24 shows chlorophyll, carotenoid, and protein bands (amide I, protein-like CH stretching) similar to EM7 (Figure 4I). The colocalization with TPF in the overlay image and correlation with TPF in the scatter plot reveal that EM24 represents the residual intact or nearly intact thylakoid structures inside the leaf (Figure 3L,M).

**Figure 5:**
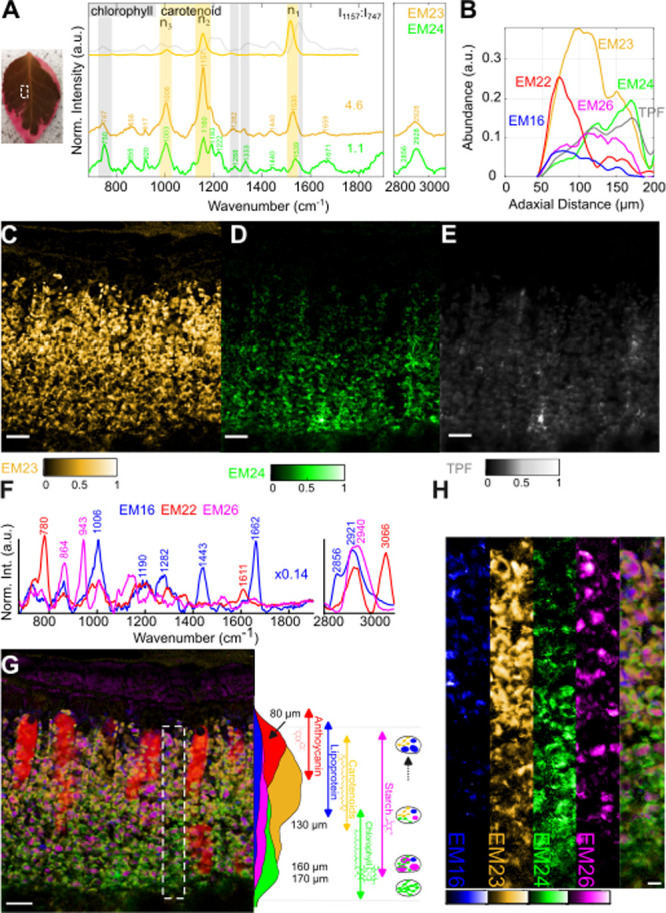
Intraleaf mesophyll gradients in a cross section of a green-red region of . (A) Endmember spectra of EM23 and EM24; (B) mean abundance profiles along the distance from the adaxial surface (3-polynom Savitzky-Golay filtered with frame length 61). EM16 abundances in cuticle and nuclei were masked to only depict plastoglobuli distribution. (C–E) Abundance maps of EM 23, EM24, and TPF; (F) Endmember spectra of EM16, EM22, and EM26 depicting lipoprotein, anthocyanin, and starch, respectively. (G) Overlay image of EM16 (blue), EM22 (red), EM23 (orange), EM24 (green), and EM26 (magenta) and mean abundance along the adaxial-abaxial axis adapted from (B). (H) Marked region in (G) with individual and overlay abundance maps. Scale bars (C–E, G): 20 μm; (H): 5 μm.

Further high light/stress response spectral features are shown in a red-green-colored leaf. Figure 5F shows spectra of the endmembers EM16, EM22, and EM26, which are unique (EM22 and EM26) or show increased abundances (EM16) in the red-green leaf as opposed to the green leaf, possibly reflecting further high light and/or stress responses. The abundances are depicted together with the chloroplast pigment spectral features EM23–24 in the overlay image in Figure 5G.

The red color in autumn or stressed leaves is usually due to an accumulation of anthocyanins, a group of flavonoid compounds, and is revealed here via EM22 with high abundances in some vacuoles of the palisade cells (Figure 5G). The spectrum shows an unusually high band at 3066 cm^–1^ and a further high band at 780 cm^–1^, which is related to aromatic CH-stretching^58^ and CH out-of-plane deformation of aromatic substituted rings,^58,59^ respectively (Figure 5F). The pronounced signals of only a few bands give rise to an electronic preresonance enhancement of the BCARS process (Figure S3). The anti-Stokes signal at 3066 cm^–1^ (∼623 nm) overlaps with a broad absorption band of cyanidin-3-glucoside, a common anthocyanin in plants, with 83 nm distance to the absorption peak maximum at 540 nm (Figure 1D). The CH out-of-plane deformation of aromatic substituted rings also couples to the chromophore system and is thus also signal-enhanced. In plants, the flavylium cation, responsible for the color, is stabilized via copigmentation, e.g., with colorless flavonols.^60,61^ The observed aromatic ring signal enhancement might be due to copigmentation, which results in a highly ordered structure, leading to a strong coherent amplification of the π–π stacked supramolecular aromatic ring ensemble.

A further typical high light growth response is the increased production of starch in the chloroplasts depicted here by EM26, which shows a unique amylose/amylopectin skeletal mode at 943 cm^–1^.^43^ The EM26 abundance map, especially the sectioned part in Figure 5H, highlights starch deposits similar to the size of a chloroplast with a diameter of up to 5 μm. In contrast to the observed pigment gradient, these starch-rich chloroplasts are quite homogeneously distributed all over the entire mesophyll, overlapping with the intact chlorophyll-rich thylakoid structures at the abaxial side as well as the chlorophyll-degraded/carotenoid-rich thylakoid structures at the adaxial side (Figures 5B,G and S7E,F).

EM16 shows protein-specific bands^43^ (amide I, amide III, and phenylalanine band at 1662, 1282, and 1006 cm^–1^), an intense CH deformation at 1443 cm^–1^ and lipid-like stretching at 2856 cm^–1^ similar to a combination of EM10 and EM1. Like the green leaf, the abundances are increased in the cuticle and nuclei of the mesophyll (Figure S7B). However, we see further aggregates toward the adaxial side of the mesophyll, which are less distinct in the green leaf with reference to the mean abundances inside the nuclei (as a combination of EM1 and 10, see Figure S12). We hypothesize that these lipoprotein-enriched aggregates reflect plastoglobuli, whose size and number increase under high light conditions, reflecting degradation products of the thylakoid structures. In contrast to the equally distributed starch grains, the plastoglobuli clearly increase toward the adaxial side of the mesophyll. Our method clearly shows a diversity of chloroplast states, including changes associated with the sun-to-shade gradient across the leaf. The method will therefore provide a new tool to probe this aspect of metabolism.

### Calcium Oxalate Crystals

Calcium oxalate can be present in different hydration states inside plants, which can be assigned via the OH-stretching signals (Figure 6A).^62–64^ EM11–12 show a very fine OH-stretching structure with four bands at 3040, 3240, 3328, and 3405 cm^–1^, which are attributed to the monohydrate form, the most stable form. EM13–14 show less selective broad OH-stretching bands, which are attributed to the dihydrate and/or the rarest and unstable trihydrate.^64^ Inconsistencies in the FP region in comparison to the literature hinder hydrate state identification via these signals, indicating a highly complex, little-understood system so far. The overlay image of the abundances reveals that the crystals of different hydrate states can be highly superimposed in plants (Figure 6B).

**Figure 6:**
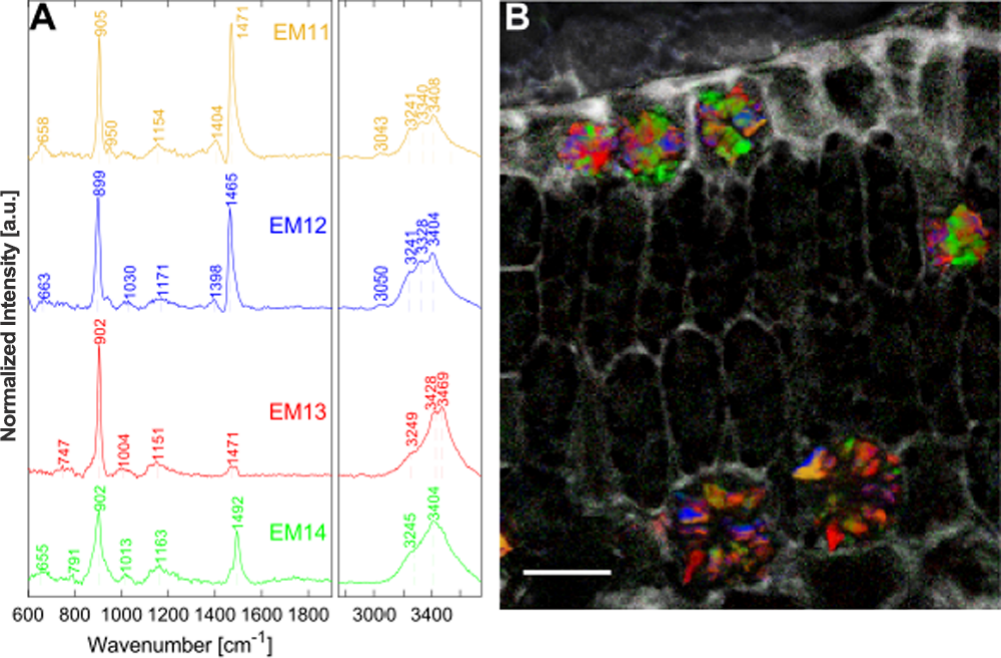
Unmixing results of the calcium oxalate crystal regions in plant leaf cross sections of including OH-stretching signals. (A) Endmember spectra. (B) Overlay image with cell walls (gray); Scale bar: 20 μm.

## Conclusions

BCARS imaging in combination with an optimized unmixing procedure gives deep insight into the chemical composition, processes, and distribution of different cellular compartments in plant leaves. The method enables high-speed chemical imaging and allows us to scan large areas of a sample with cellular to subcellular resolution, giving a system perspective on the sample, which is here exemplarily elucidated on structural-chemical heterogeneous leaf cross sections, enabling the characterization of intraleaf photosystem gradients up to the accumulation of specific secondary metabolites. This system view might be of great value in plant physiology, biochemistry, and plant disease characterization. Agrochemical and environmental research could profit from the capability to simultaneously visualize the uptake and effects of a compound in a plant system. We demonstrated that we can extract molecular vibrational-rich information from the photosystem regions, which in combination with the accompanying chlorophyll fluorescence extracted from the BCARS raw data gives deep insights into intraleaf gradients, possibly making it an outstanding imaging tool for photosynthesis research in particular. Thanks to the high-speed spectral acquisition capabilities, three-dimensional chemical imaging or time-resolved studies could also be easily performed.

## Supplementary Material



## Notes

Emission spectra of a plant sample (Figure S1); energy diagrams of fluorescence phenomena (Figure S2); possible electronic resonance and electronic preresonance (Figure S3); five repeated runs of the endmember extraction (Figure S4); MaxD followed by FCNNLS unmixing (Figure S5); similarity-analysis of the [5 5] neighborhood pixel spectra (Figure S6); pixel neighborhood (Figure S7); Raw spectra generated from corresponding endmember pixels (Figure S8); green-red region of a plant leaf cross section of (Figure S9); BCARS data (Figure S10); background signals (Figure S11); lipoprotein features (Figure S12); photographs of red (left) and green (right) leaves (Figure S13); and Raman-like bands obtained from BCARS (Table S1) (PDF)
