# Supplementary material for: Chemical Fingerprint Imaging In Planta with Broadband Coherent Anti-Stokes Raman Scattering Microscopy
Source: Anal Chem. 2025 Jul 28;97(31):16868–76. doi: 10.1021/acs.analchem.5c01980 (PMC12355479; doi:10.1021/acs.analchem.5c01980)
Supplement: Supplementary file 1 [file ac5c01980_si_001.pdf]

Supporting Information

Chemical Imaging of Plant Leaves with Broadband Coherent Anti-Stokes Raman-Scattering Microscopy

Paul Ebersbach[1], Nicholas Smirnoff[2], Charles H. Camp Jr.[3], and Julian Moger[1]*

[1]School of Physics, University of Exeter, Exeter EX4 4QL, United Kingdom
[2]Biosciences, University of Exeter, Exeter EX4 4QL, United Kingdom
[3]Biosystems and Biomaterials Division, National Institute of Standards and Technology, 100 Bureau Dr., Gaithersburg, MD 20899, USA

Origin of absorption and fluorescence phenomena in chlorophyll molecules

Figure S1 shows emission spectra of a plant leaf sample with the single pump and stokes beam excitation and in combination. The combined action of the pump and stokes beam generates a broad peak at 680 nm superimposing the Nonresonant-Background. This peak can be attributed to chlorophyll fluorescence and is also observable with single pump beam excitation with similar high intensity and with single stokes beam excitation with lower intensity.

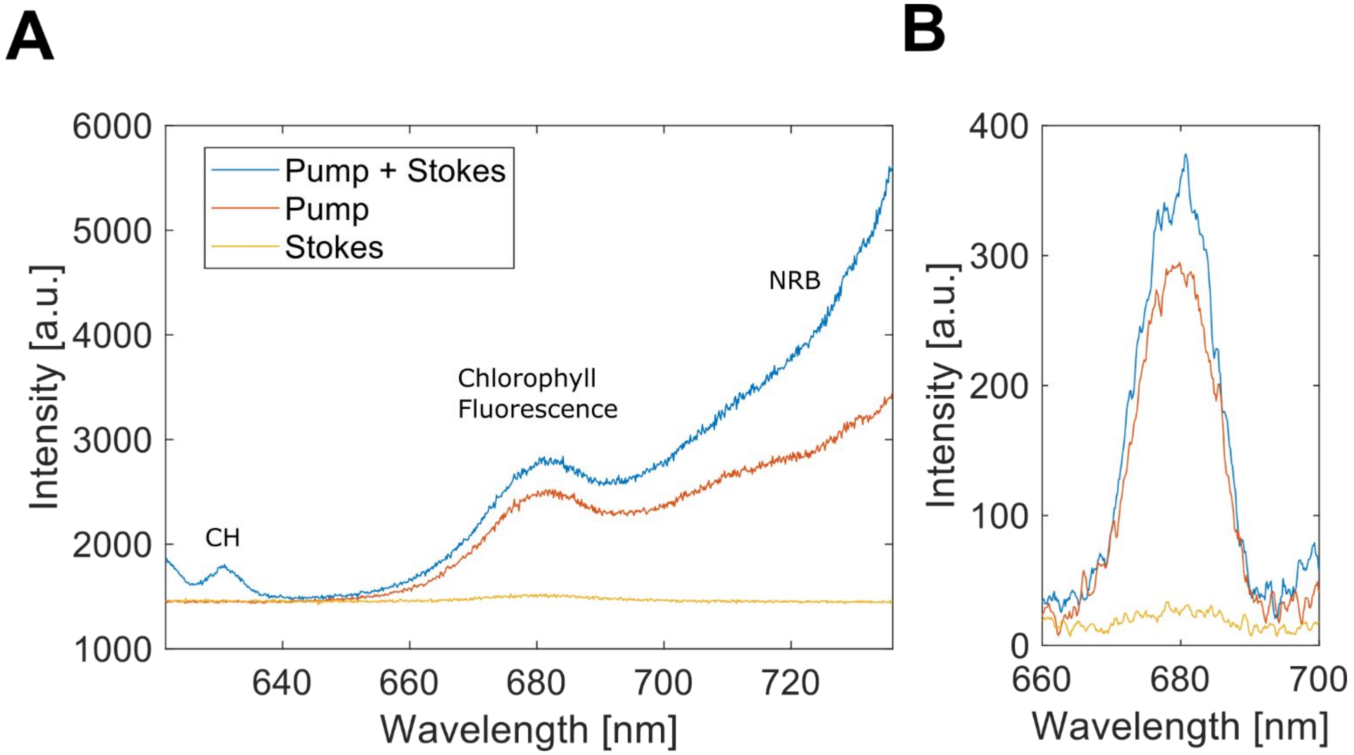

Figure S1. Emission spectra of a plant sample excited with the pump beam at 770 nm and stokes beam between 900 – 1350 nm. (A) Raw spectra (B) Baseline corrected[1] spectra clarifying the intensity of the chlorophyll fluorescence peak.

Chlorophyll a and b show several absorption peaks in the red range between 600–700 nm and in blue spectral range between 400–500 nm, called the Q_y and Soret absorption bands, respectively (Fig 1 E). The wavelength of the pump at 770 nm or stokes at 900–1350 nm are far away from these electronic transitions. One-photon absorption is therefore not likely. However multiphoton processes may excite from the ground state S_0 to the electronic excited singlet states.

The observed single pump excited fluorescence phenomenon can be attributed to a two-photon absorption process to an Soret state, which is similar to an one-photon absorption process at double energy at 385 nm and is followed by two-photon fluorescence emission (Fig. S2 A). The similar high fluorescence intensity of the single pump excitation and the combined pump and stokes excitation hints that the two-photon absorption of the pump beam is the dominating process for the fluorescence emission. Further contributions might be due to the combined action of the pump beam and stokes beam with a possible two-photon absorption between 450 – 490 nm and the two-photon absorption through the stokes beam at the highest energies of the broad beam (Fig. S2 B and C). The obtained low fluorescence intensity via single stokes excitation hints that this process has only a minor contribution (see Fig S1 B).

Electronic transitions to the Q_y absorption bands might be obtained in the two-colour BCARS and/or three-colour BCARS process (Fig. S2 D and E) or via an already emitted BCARS signal in a second molecule (Fig. S2 F). The maximum absorbance in chlorophyll a (661 nm) or b (643 nm) would be obtained when the two-colour, three-colour BCARS process or generated BCARS signal matches the nonresonant vibrational region at 2140 cm^{-1} or 2590 cm^{-1}, respectively. The fingerprint region (<1800 cm^{-1}) is just below the maximum chlorophyll a and b absorbance, while the CH stretching is just above that region of highest absorbance. Some overlapping with the highest energy levels of the chlorophyl b molecule might still result in the absorbance of some vibrational contrast containing signals in the CH stretching region (~630 m).

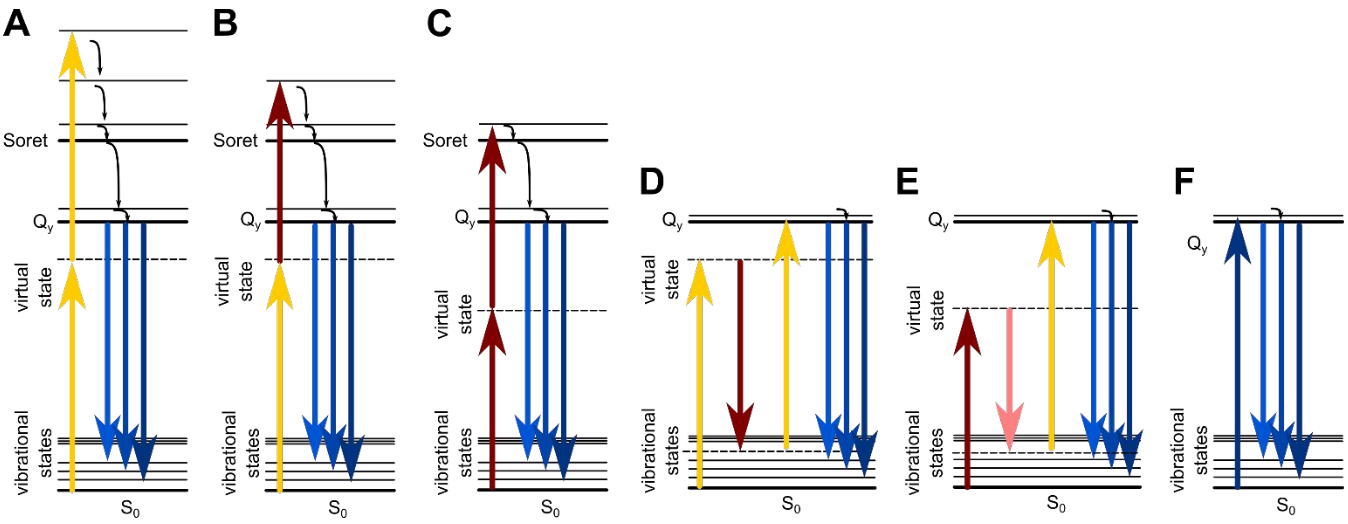

Figure S2. Energy diagrams of fluorescence phenomena of chlorophyll a and b. (A) Two-photon absorption of the pump beam followed by two-photon fluorescence (B) Two-photon absorption of the pump and stokes beam followed by two-photon fluorescence (C) Two-photon absorption of the stokes beam followed by two-photon fluorescence (D) Two-colour excitation induced fluorescence via a nonresonant vibrational state between the CH stretching and fingerprint region (E) Three-colour excitation induced fluorescence via a nonresonant vibrational state between the CH stretching and fingerprint region (F) One-photon absorption of BCARS light followed by fluorescence emission.

Electronic Resonance and Electronic Preresonance broadband coherent Anti-Stokes Raman-scattering (ER-BCARS and EPR-BCARS)

A CARS process becomes electronic resonant when at least one of the laser beams and/or the generated anti-Stokes signal matches an electronic transition in the excited molecule[2]. As the pump beam at 750 nm and stokes beam at 900-1350 nm are far away from any electronic transition in plant pigments like chlorophyll a and b, anthocyanins and carotenoids direct electronic excitation with one of the laser beams is not likely (Fig 1 E).

However at least electronic preresonance enhancement may result from the laser beams or the BCARS signals, whose wavelengths are brought due to the blue-shift closer to the absorption peaks of the pigments. Wei et al.[3] defined an electronic preresonance window, in between the rigorous resonance and the nonresonance regions for Electronic Preresonance Stimulated Raman Scattering: In this window the pump laser frequency (or in our case also the stokes beam and/or anti-stokes signal) should be 2-6 Γ_e away from the molecular absorption maximum (Γ_e is the homogeneous line width of the electronic transition, ~700 cm^{-1}). The two boundaries are set to ensure a fine balance between achievable EPR-Raman enhancement and fine chemical selectivity with a sufficiently attenuated electronic background[3].

First of all the unusual high signal intensity of the aromatic CH stretching at 3066 cm^{-1} obtained in some palisade cell vacuoles of the red leaf cross section attributed to an anthocyanin accumulation give rise to an signal enhancement effect. The corresponding anti-Stokes signal at 623 nm overlaps with the broad absorption band of the anthocyanin cyanidin-3-glucoside with 83 nm distance to the absorption peak maximum at 540 nm. According to the presonance window calculated here with 584 – 698 nm ($\Gamma_e \sim$ 700 cm^{-1}) the electronic preresonance condition is fulfilled for the anti-Stokes signal at 623 nm (the pump beam stokes beam are outside the preresonance window). Thus a preresonance enhancement might be a plausible enhancement mechanism here (Fig. S3). And indeed the only signal enhancement of a few aromatic ring specific vibrations coupled to the chromophor group, a well know condition for electronic resonance (or preresonance), hint that the signal enhancement is due to EPR-BCARS.

A high signal intensity was also obtained for the photosystem specific signals (combination of chlorophyll and carotenoid bands) enabling high contrast imaging of the chloroplasts in the palisade cells indicating a further underlying signal enhancement mechanism.

For the Q_y band in Chlorophyll a (Max 661 nm; preresonance window: ~700 – 790 nm and ~570 – 627 nm with $\Gamma_e \sim$ 400 cm^{-1} [4]) the preresonance condition is fulfilled for the pump beam and anti-Stokes signals < 1300 cm^{-1}. Anti-Stokes Signals between 1300 – 2960 cm^{-1} have energies closer to the absorption band then the defined preresonance window. These signals are located in the resonance region. For the Q_y band in Chlorophyll b (Max 643 nm; preresonance window: ~ 680 – 760 nm and ~ 557 – 612 nm with $\Gamma_e \sim$ 400 cm^{-1} [4]) both the pump and stokes beam are located in the nonresonance region . Anti-Stokes signals < 1760 cm^{-1} are located in the preresonance region and anti-Stokes signals 1760-3380 cm^{-1} are located in the resonance region. To sum up it can be said that also for chlorophyll a and b preresonance enhancement of the BCARS signals is a plausible enhancement mechanism (Figure S3 B and C).

The resonance-Raman-like high signal intensity of only a few carotenoid specific bands might lead to the conclusion that the selective enhancement of these signals is also due to a ER-BCARS or EPR-BCARS process. However carotenoids only absorb blue light far away from the laser beam and anti-Stokes energy levels. Electronic resonance or preresonance (beta-carotene highest energy local max at 477 nm; preresonance window: ~ 510 – 600 nm with $\Gamma_e \sim$ 700 cm^{-1}) is neither fulfilled for the pump and stokes beam nor for the anti-Stokes signals. Unusual high signal responses in the nonresonant regions are well known for carotenoids and have been previously observed in the near-IR[5] but also via CARS[2] (Pump 915 nm, Stokes: 950 – 1200 nm). The authors propose that this phenomenon might be attributed to π-
electron−phonon-coupling firstly proposed by Castiglioni et al.[6] rather than to traditional resonance enhancement. However the CARS study[2] points out that for the nonlinear optical case its relevance is unclear and has to be investigated in greater detail. At least our consistent results with their study further hint that there must be a wavelength-independent underlying enhancement mechanism also under nonlinear optical conditions.

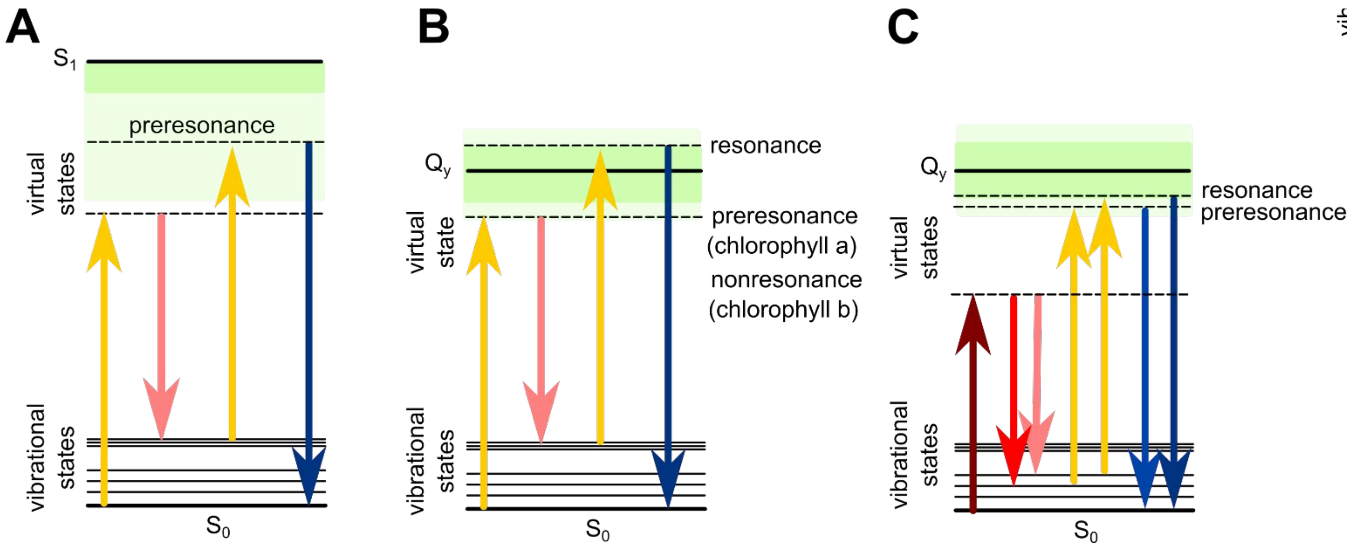

Figure S3. Energy diagrams of possible electronic resonance and electronic preresonance BCARS processes. (A) Two-colour EPR-BCARS excitation of the aromatic CH stretching in anthocyanins (B) Two-colour ER-BCARS excitation of the CH stretching in chlorophyll a and b (C) Three-colour ER-BCARS excitation of fingerprint signals at higher energy level and EPR-BCARS excitation of fingerprint signals at lower energy level in chlorophyll a and b.

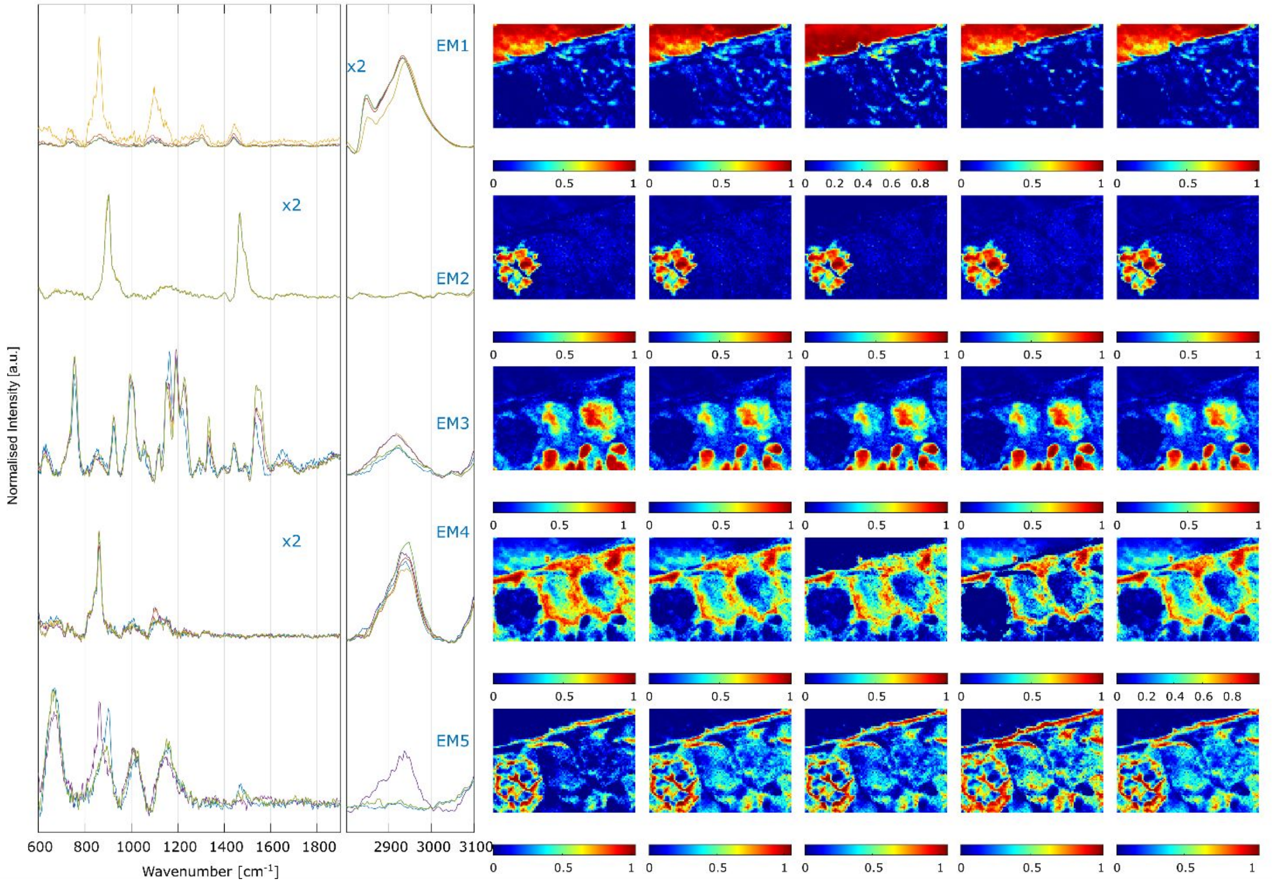

Figure S4. Five repeated runs of the Endmember Extraction via VCA followed by FCNNLS unmixing. The VCA endmember extraction algorithm shows a certain randomness which reveals here in spectral deviations in the Endmember spectra and thus small intensity variations in the abundance maps retrieved via FCNNLS unmixing.

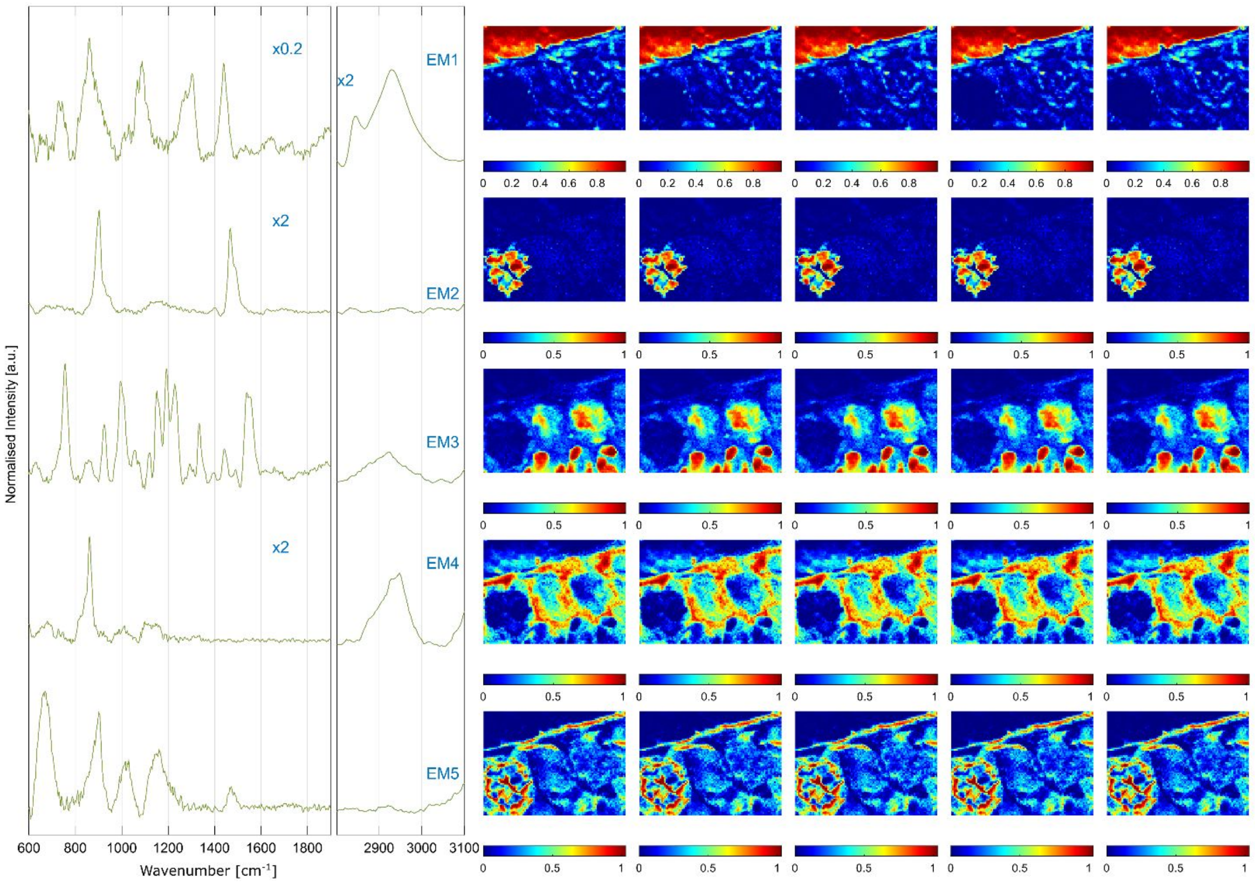

Figure S5. Five repeated runs of the Endmember Extraction via MaxD followed by FCNNLS unmixing. The MaxD endmember extraction algorithm is deterministic and gives each time the same results.

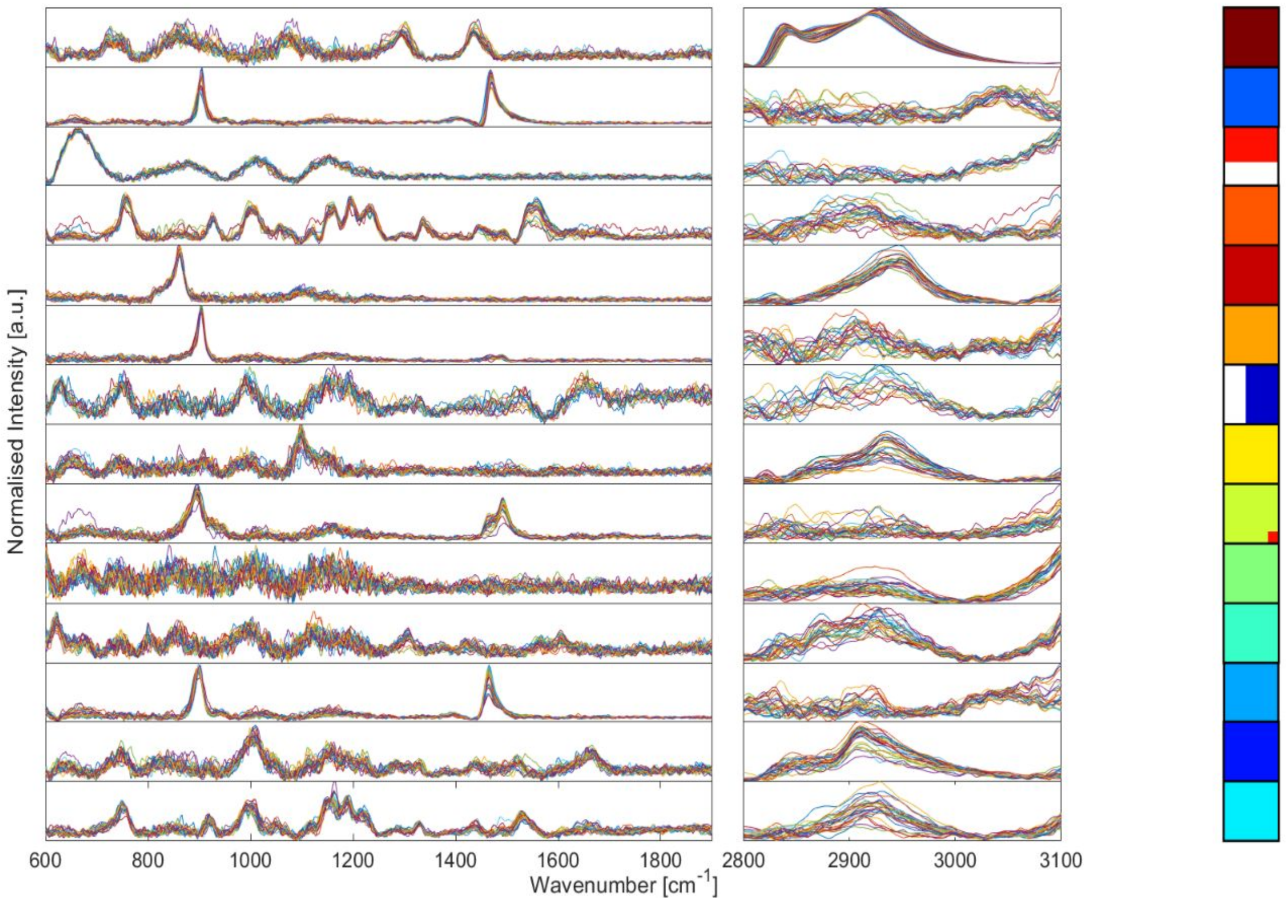

Figure S6. Similarity-Analysis of the [5 5] neighborhood pixel spectra of the median filtered Endmember superpixel spectra. The [5 5] neighborhood spectra are similar to each other and show only minor deviations. The Similarity-Analysis (hierarchical clustering with Ward-Linkage and 15 Clusters) shows that each [5 5] neighborhood pixel group builds it's own cluster (colour coded squares, each consisting of 5x5 pixels). Almost no mixing is observed between the [5 5] neighborhoods pixel spectra. Thus the superpixel spectra are "pure" superpixel spectra. The white areas are "empty" pixels in two of the superpixels located at the edge of the image.

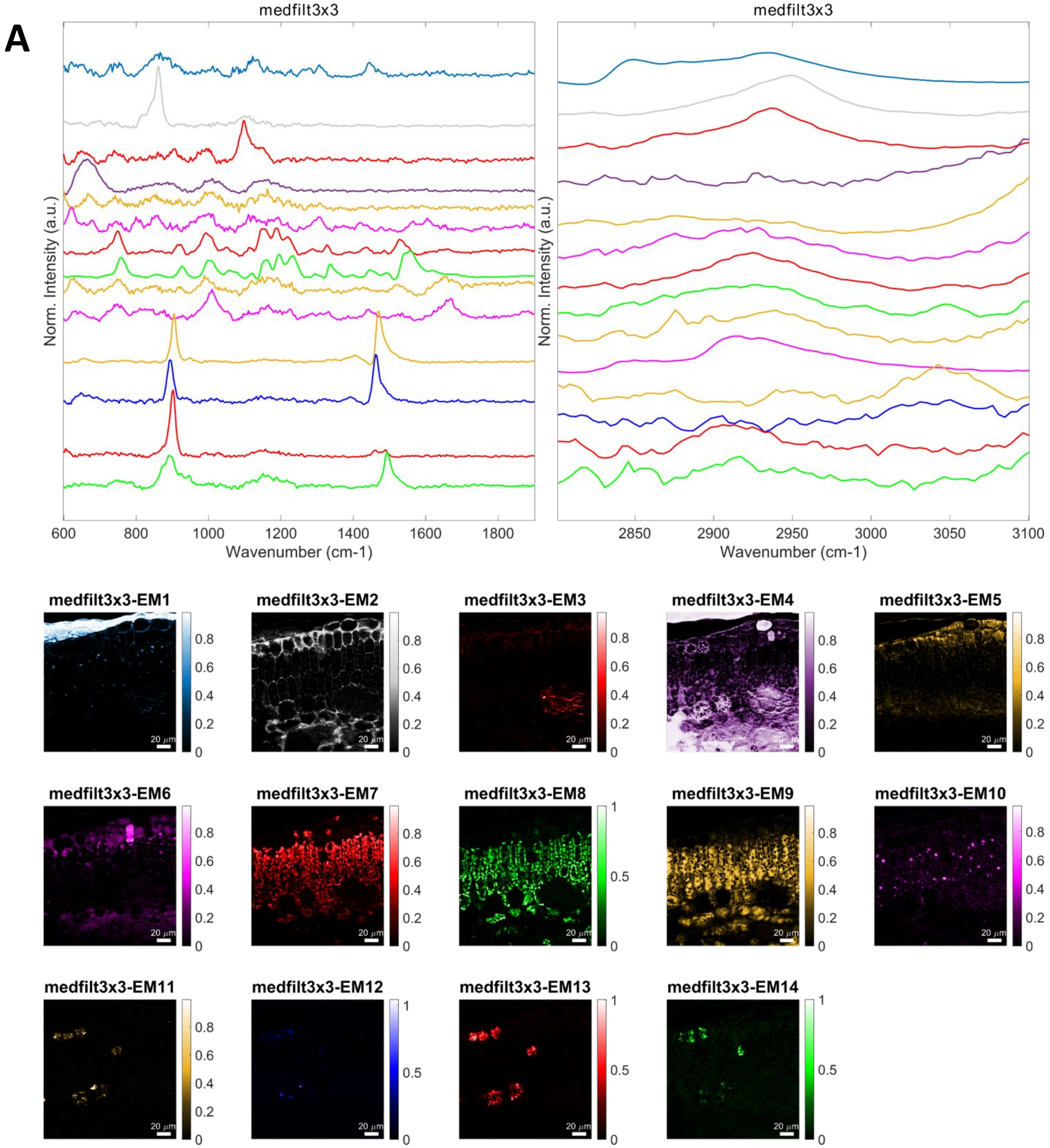
A
medfilt3x3
medfilt3x3
Norm. Intensity (a.u.)
Wavenumber (cm-1)
600
800
1000
1200
1400
1600
1800
2850
2900
2950
3000
3050
3100
medfilt3x3-EM1
medfilt3x3-EM2
medfilt3x3-EM3
medfilt3x3-EM4
medfilt3x3-EM5
medfilt3x3-EM6
medfilt3x3-EM7
medfilt3x3-EM8
medfilt3x3-EM9
medfilt3x3-EM10
medfilt3x3-EM11
medfilt3x3-EM12
medfilt3x3-EM13
medfilt3x3-EM14
20 μm

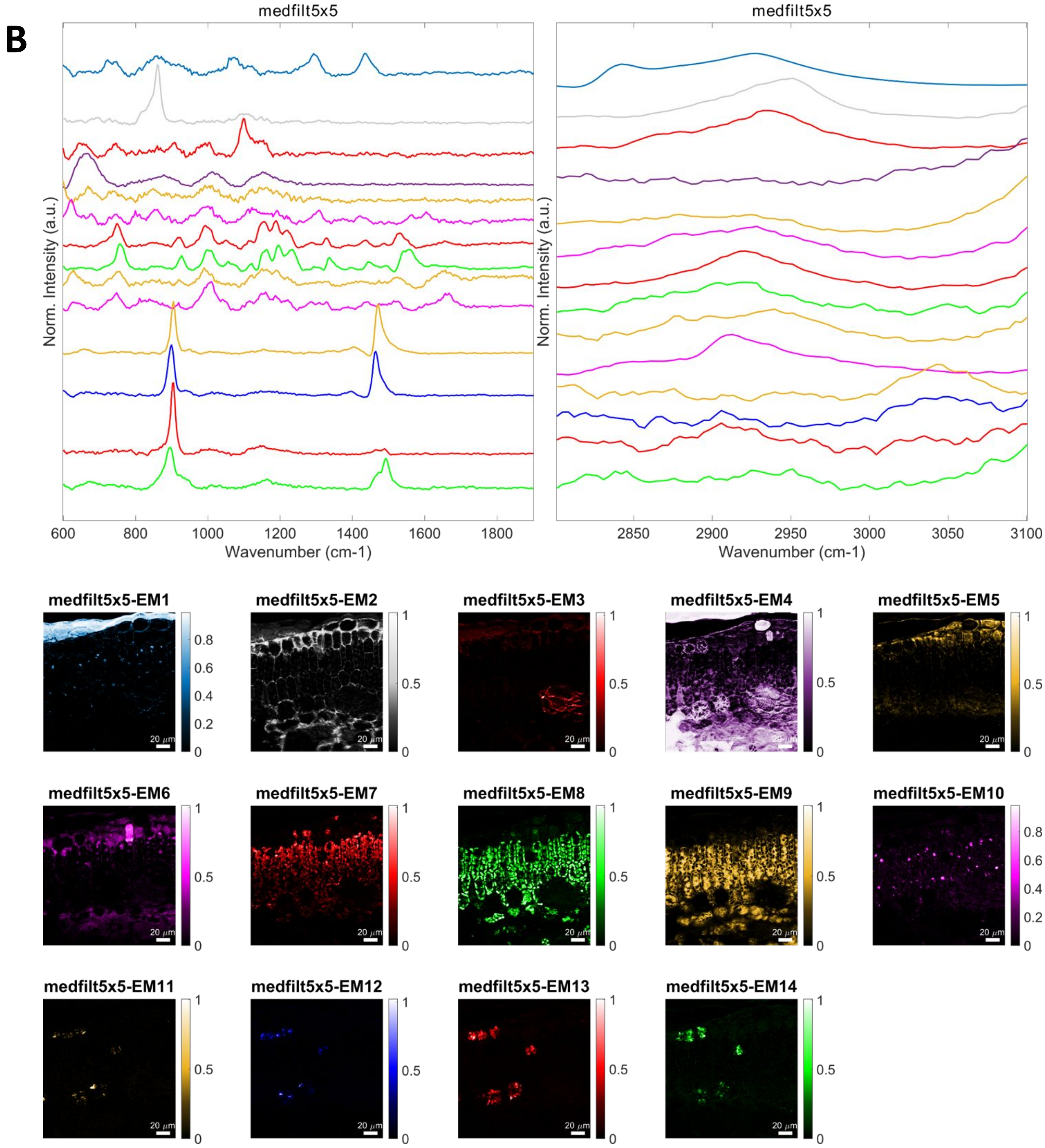
B
medfilt5x5
medfilt5x5
Norm. Intensity (a.u.)
Wavenumber (cm-1)
600
800
1000
1200
1400
1600
1800
2850
2900
2950
3000
3050
3100
medfilt5x5-EM1
medfilt5x5-EM2
medfilt5x5-EM3
medfilt5x5-EM4
medfilt5x5-EM5
medfilt5x5-EM6
medfilt5x5-EM7
medfilt5x5-EM8
medfilt5x5-EM9
medfilt5x5-EM10
medfilt5x5-EM11
medfilt5x5-EM12
medfilt5x5-EM13
medfilt5x5-EM14
20 μm

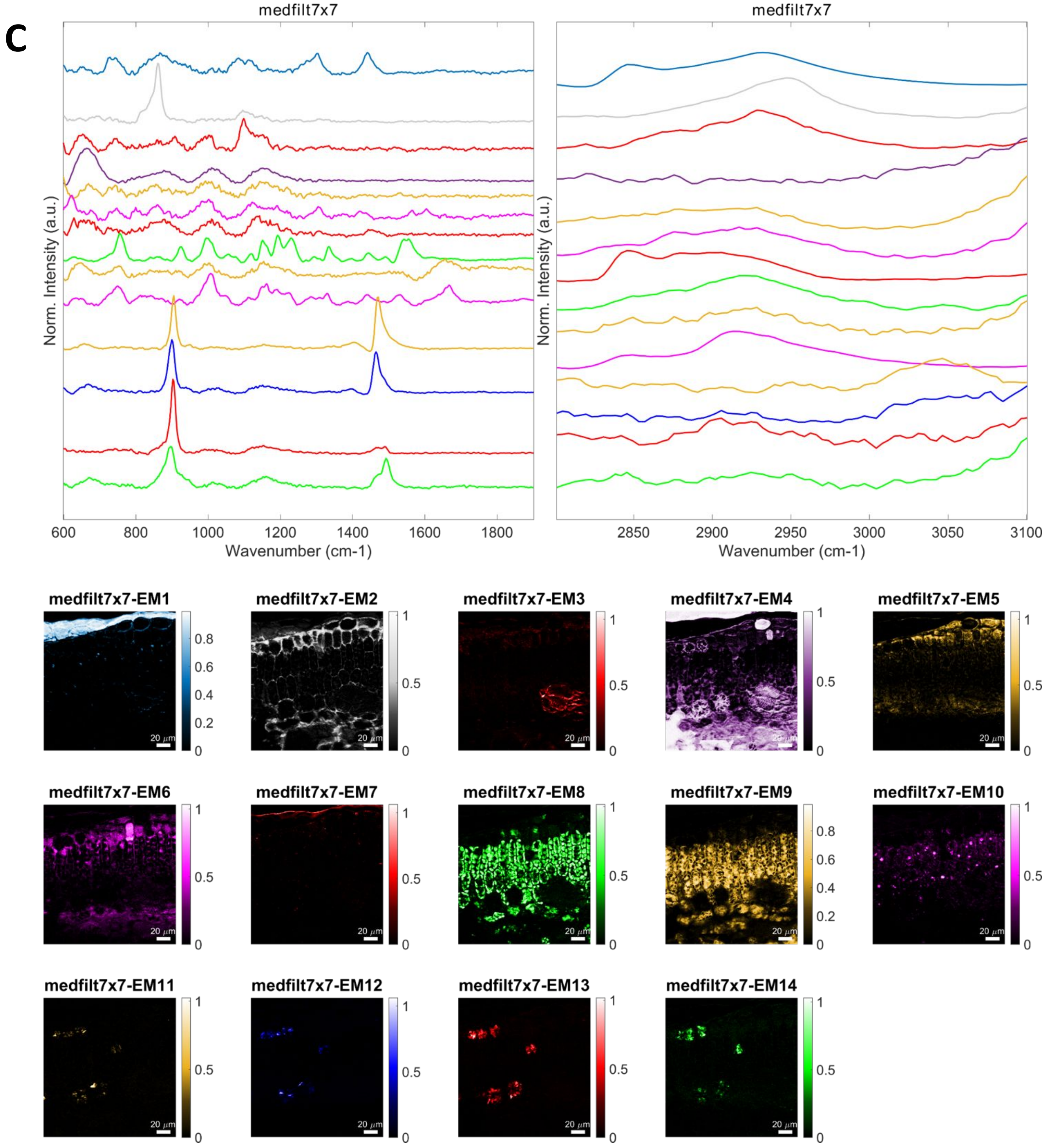

Figure S7. Comparison of unmixing with (A) 3x3, (B) 5x5 and (C) 7x7 pixel neighborhood. The 5x5 2D median filtered endmember model gives better image contrast and better spectral resolution due to the stronger averaging. The 7x7 2D median filtered endmember unmixing model extracted only two endmember from the chloroplast pigment regions instead of three thus less depicting the spectral variability in those regions. Instead it extracted a second endmember from the cuticle region.

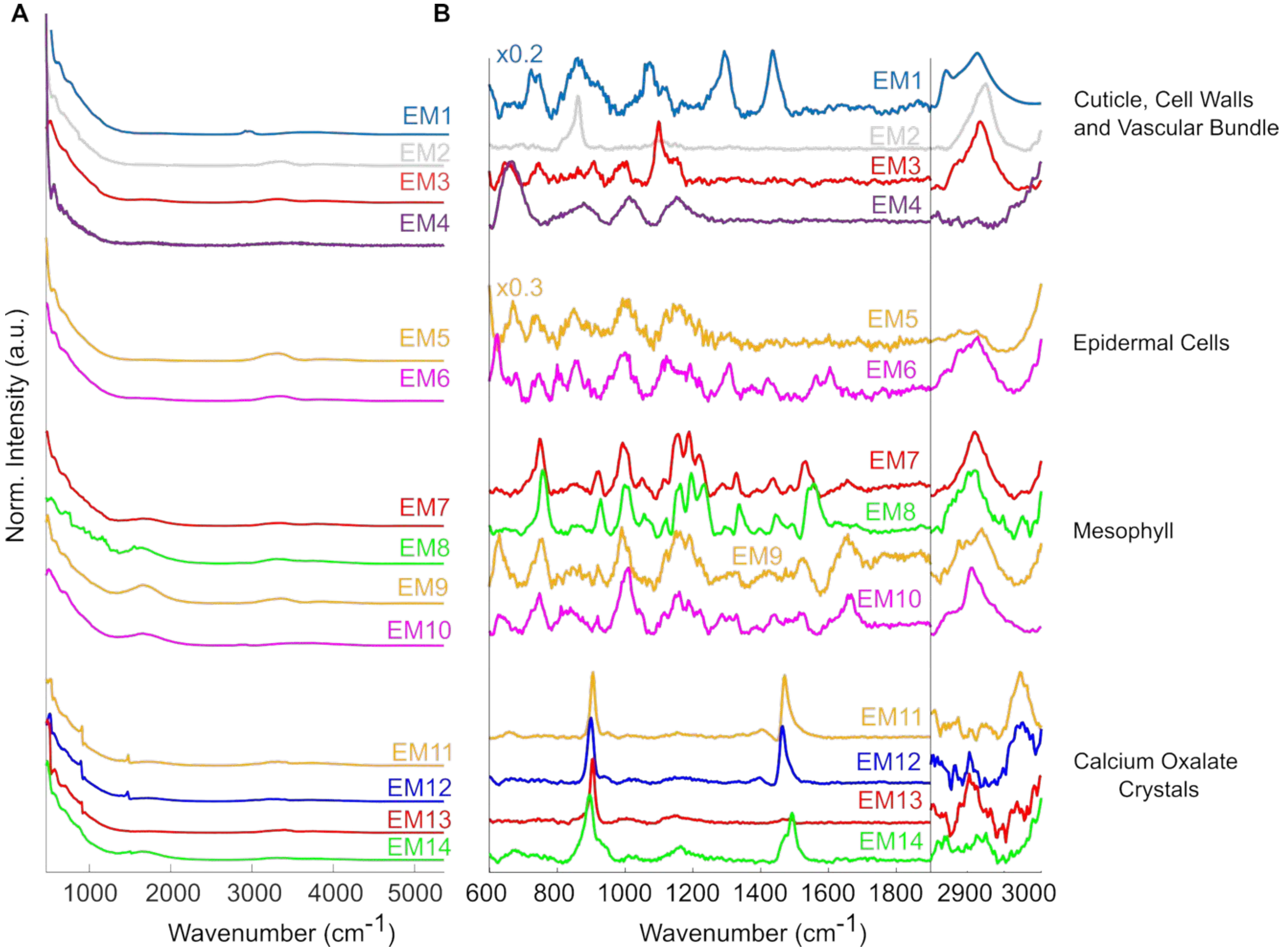

Figure S8: EM_PRspectra_vs_RawSpectra_onlySVDanscdenoised Raw spectra generated from corresponding endmember pixels after unmixing.

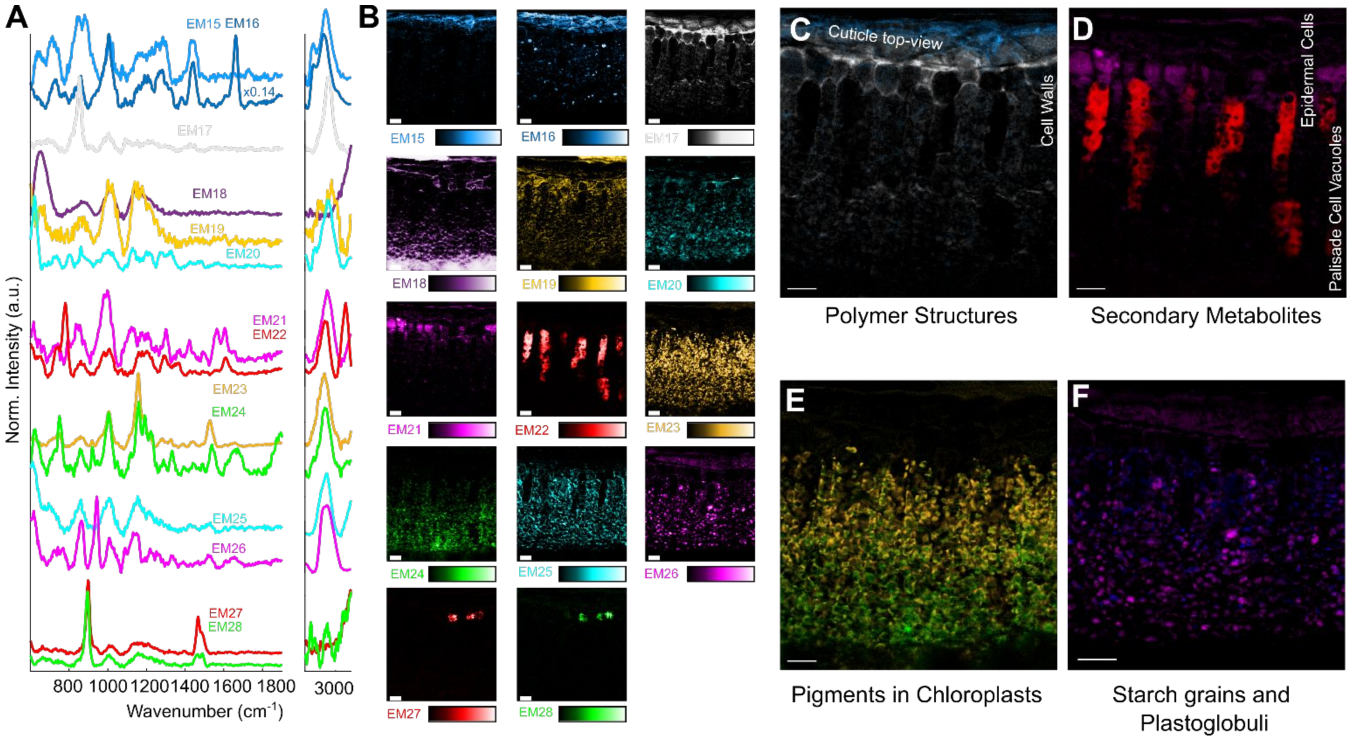

Figure S9. Unmixing results of a green-red region of a plant leaf cross section of *Euonymus fortunei*. The endmember numeration continues the endmember counting of the unmixing in Figure 3 (14 Endmembers). (A) Endmember spectra; (B) Abundance maps; (C); Overlay of EM12 (cell walls) and EM15 (lipid in cuticle); (D): Overlay of EM21 (anthocyanin) and EM2 (flavonol) depicting Secondary Metabolite accumulation. (E) Overlay of EM23 and EM24 depicting pigment spectral feature gradient in chloroplasts. (F) Overlay of EM26 (starch) and EM16 (lipoprotein). Lipoprotein-enriched nuclei in the mesophyll and cuticle were removed from the EM16 abundance map to only quantify lipoprotein-enriched plastoglobuli distribution in mesophyll (see Figure S10). EM15: Lipid in cuticle; EM16: Lipoprotein in cuticle, nuclei and plastoglobuli; EM17: Pectin in cell walls; EM18-19: Water and background signals; EM20: Lipid-like signals with high abundance in cell wall region; EM21: Flavonol in upper epidermis; EM22: Anthocyanin palisade cell vacuoles; EM23: Degraded photosystem with high carotenoid:chlorophyll signal ratio; EM24: Degraded photosystem with medium carotenoid:chlorophyll signal ratio; EM25: CH-stretching rich signals in photosystem; EM26: Amylose/amylopectin from starch with unique skeletal mode at 943 cm^{-1} [7]; EM27-28: Calcium oxalate in upper epidermis; Scalebars: 20 µm.

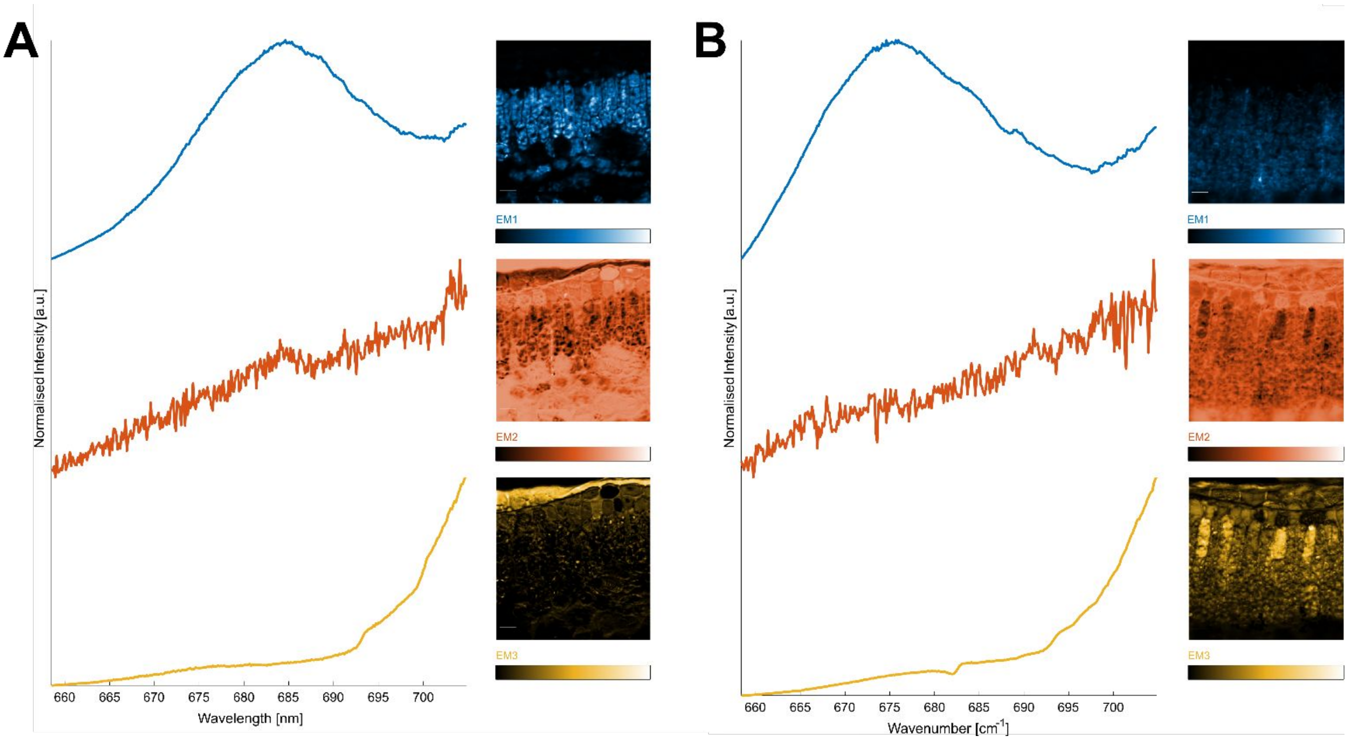

Figure S10. Unmixing results of denoised BCARS data prior to the phase retrieval step of a plant leaf cross sections of Euonymus fortunei. The spectral range was restricted towards 659 – 705 nm (1200 – 2200 cm^{-1}). (A) Green region; (B) Green-red region; EM1: Two-Photon Fluorescence of Chlorophyll a with peak maximum at 685 nm (A) and 676 nm (B); EM2: Background signals; EM3: Nonresonant Background; Scalebars: 20 µm.

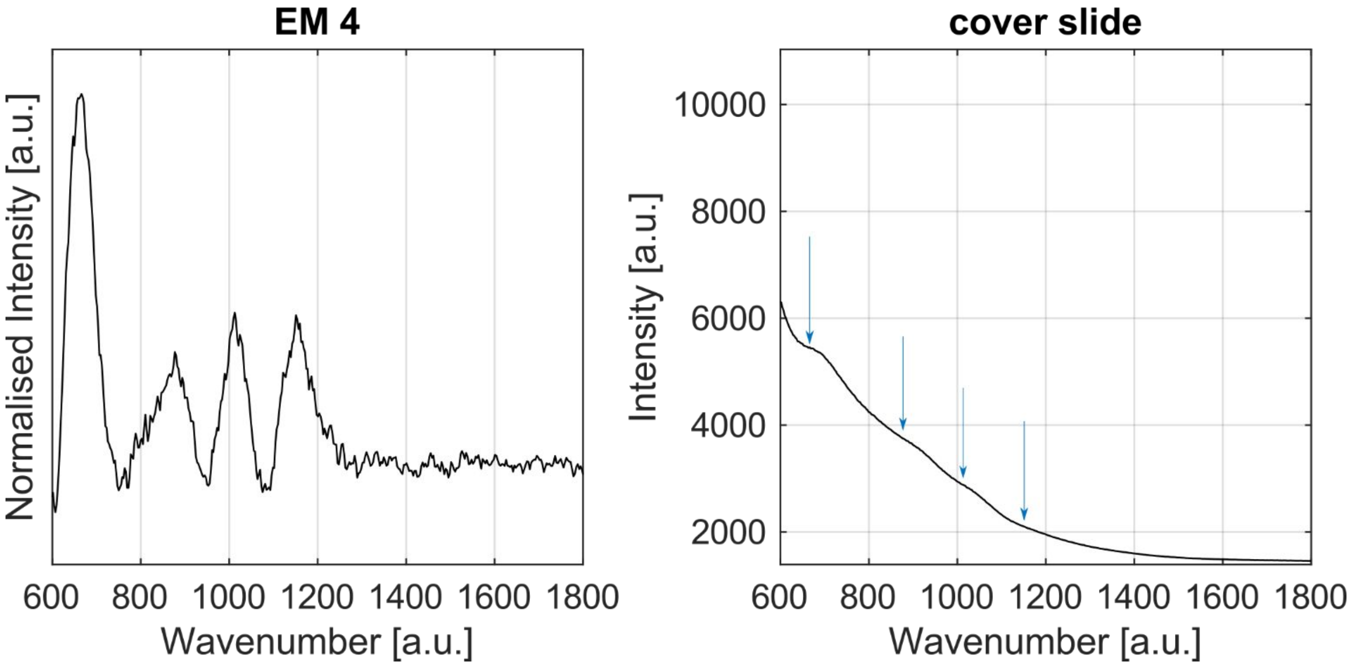

Figure S11. Origin of the background signals obtained in EM4. The raw BCARS spectrum of a cover slide reveals that the signals originate from the cover slide.

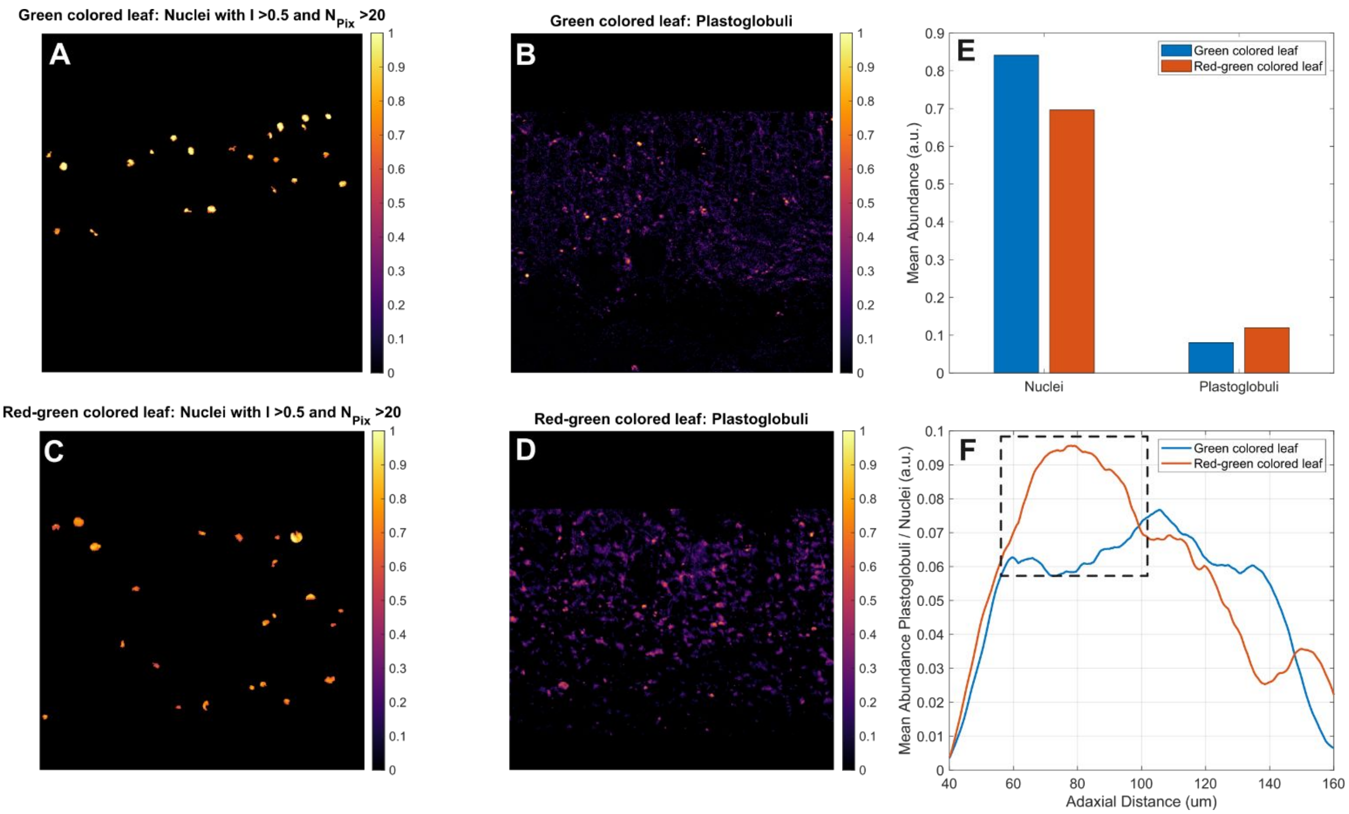

Figure S12. Separation of lipoprotein features into nuclei and plastoglobuli in a green and green-red colored leaf cross section of Euonymus fortunei. The lipoprotein map of the green leaf was calculated as a mean from EM1 (lipid-rich) and EM10 (protein-rich). The lipoprotein map of the green-red leaf is EM16. (A, C) Nuclei were defined as quite pure regions with I > 0.5 and a pixel number size > 20. Note that some of the large and bright features might be highly plastoglobuli-enriched gerontoplasts in a late state consisting of almost pure lipoprotein. These cannot distinguished here from the nuclei. (B, D) Plastoglobuli are depicted by the residual features. Note that abundances from the cuticle were manually masked. (E) Mean abundances in nuclei and plastoglobuli (F) Mean Abundance profiles of the plastoglobuli map along the distance from the adaxial surface (3-polynom Savitzky-Golay filtered with frame length 61). The mean abundances were normalised via the mean abundances in the nuclei. The red-green colored leaf shows increased abundances of plastoglobuli towards the adaxial side opposed to the green colored leaf (marked via the rectangle).

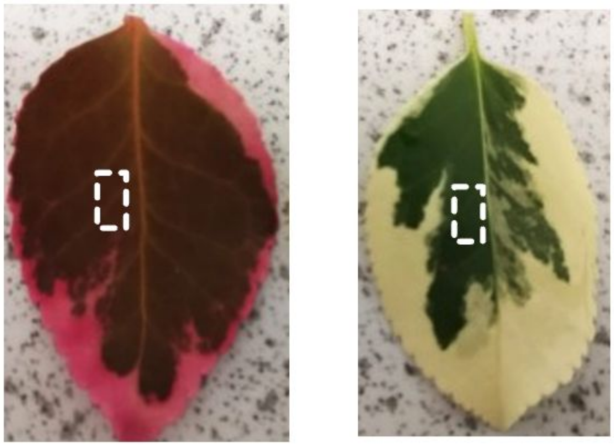

Figure S13. Photographs of red (left) and green (right) leaves used in the study and the regions where sections where taken.

Table S1. Assignment of the Raman-like bands obtained from BCARS of the green region of a Euonymus fortunei leaf cross section with Spontaneous Raman bands from the literature. ν: stretching; δ: deformation; r: rocking, w: wagging, ip: in plane

	BCARS [cm^{-1}]	Assignment	Raman [cm^{-1}]
Cuticle and Cell Walls	723	δ (CH2) rocking[8]	723 (IR)[8]
	860	α-Glycosidic bond in pectin[9]	866[9]
	1073	ν(CO) + δ(OH) in pectin[10]	1074[9]
	1096	ν asym (C-O-C) in cellulose[7]	1094[7]
	1110	ν(CC)(CO) in pectin[10]	
	1154	ν sym (C-O-C) in cellulose[7]	1122[7]
	1168	Ring δ ip (CH), ν (C-O-C) ester[9]	1167[9]
	1293	δ(CH2) twisting saturated wax[9]	1298[9]
	1434	δ(CH2) lipids[9]	1438[9]
	1601	Aromatic ν(C=C) phenolic compound[9]	1604[9]
	1639	Unsaturated ν(C=C) phenolic compound / coniferyl aldehyde[9]	1632[9]
	1721	ν(C=O) cuticular wax[9]	1727[9]
Flavonol	623	δ, all rings[11]	630 (IR)[11]
	799	ω(CH) + breathing at dihydroxyphenyl ring[12]	794[12]
	852	ν(CC) in glycopyranosyl ring[11]	850[11]
	891	ω(CH) at dihydroxyphenyl and mannopyranosyl rings and between mannoyranosyl and glucopyranosyl rings[11]	899[11]
	1001	δ(C-CC) + ν(CC) in dihydroxyphenyl[12]	998[12]
	1122		1114
	1192	δ(C-C-H) in benzene and dihydroxyphenyl rings	1183 (IR)
	1307	δ(C-C-H) in all rings + r(C-H) at all rings	1307 (IR)
	1374	r(C-H) at mannopyranosyl ring	1369
	1422		1422[13]
	1564	ν(C=C) in benzene and dihydroxyphenyl rings[12]	1560[12]
	1604	ν(C=C) in benzene, heterocyclic and dihydroxyphenyl rings[12]	1606[12]
	1654	ν(C=O) at heterocyclic ring[12]	1658[12]
Photosynthetic Pigments	747-756	most characteristic chlorophyll band[7], ip δ pyrrole ring[14]	740[7], 746[14]
	922-928		
	990-1010	ip r(CH3) coupled with C-C bonds of polyene chain (ν3 band of carotenoids)[15]	1000-1020[15]
	1050-1055	conformation sensitive mode R8[16]	1045[16]
	1150-1250	in-phase ν(C-C) of polyene chain, (ν_2 band of carotenoids)[15]	1150-1170[15]
	1287-1295	most characteristic chlorophyll band[7]	1286[7]
	1328-1337	most characteristic chlorophyll band[7]	1326[7]
	1389-1395		1390
	1437-1443	conformation sensitive mode R6[16]	1440[16]
	1489-1492		
	1530-1542	in-phase ν(C=C) of polyene chain, (ν_1 band of carotenoids)[15]	1500-1550[15]
	1550-1557	vibrational modes in chlorin ring (chl a 5-coordinated) [16]	1555[16]
	1630	ν(HC=O) in chl b[17]	1630[17]
	1654	amide I[7]	1655[7]

	1683	ν(C=O) in chl a[17]	1659-1685[17]
Anthocyanin	780	CH out-of-plane δ aromatic substituted ring[18,19]	780[18,19]
	3066	aromatic CH stretching[18]	3062[18]
Starch	943	amylose/amylopectin skeletal modes[7]	941[7]
Lipoprotein	1006	δ(ring) Phenylalanine[7]	1006[7]
	1282	amide III[7]	>1275 (α-Helix)[7]
	1443	$\delta(CH_2)$ [7]	1444[7]
	1662	amide I[7]	1655-1685[7]
	2856	ν_s (CH_2) [7]	2850[7]
Calcium Oxalate	1471-1492	ν sym (C-O)[20]	1472-1490[20] /1468[21]
	899-905	ν(C-C) + in-phase sym δ(OCO)[20]	896-912[20] / 909[21]
	3045		3056[20] / 3067[21]
	3241		3256[20] / 3248[21]
	3335		3340[20] / 3359[21]
	3406	ν(OH) in monohydrate[20,21]	3426[20] / 3462[21]
	3250		~3250[20] / 3266[21]
	3420	ν(OH) in dihydrate[20,21]	~3430[20] / 3467[21]
	3245		~3200[20]
	3470	ν(OH) in trihydrate[20]	~3500[20]

References

(1) Eilers, P.; Boelens, H. Baseline Correction with Asymmetric Least Squares Smoothing. *Unpubl. Manuscr* **2005**.

(2) Slepkov, A. D.; Barlow, A. M.; Ridsdale, A.; McGinn, P. J.; Stolow, A. In Vivo Hyperspectral CARS and FWM Microscopy of Carotenoid Accumulation in H. Pluvialis. In *Multimodal Biomedical Imaging IX*; International Society for Optics and Photonics, 2014; Vol. 8937, p 893709.

(3) Wei, L.; Min, W. Electronic Preresonance Stimulated Raman Scattering Microscopy. *J. Phys. Chem. Lett.* **2018**, *9* (15), 4294–4301.

(4) Saito, K.; Suzuki, T.; Ishikita, H. Absorption-Energy Calculations of Chlorophyll a and b with an Explicit Solvent Model. *J. Photochem. Photobiol. A Chem.* **2018**, *358*, 422–431.

(5) Parker, S. F.; Tavender, S. M.; Dixon, N. M.; Herman, H.; Williams, K. P. J.; Maddams, W. F. Raman Spectrum of β-Carotene Using Laser Lines from Green (514.5 Nm) to near-Infrared (1064 Nm): Implications for the Characterization of Conjugated Polyenes. *Appl. Spectrosc.* **1999**, *53* (1), 86–91.

(6) Castiglioni, C.; Del Zoppo, M.; Zerbi, G. Vibrational Raman Spectroscopy of Polyconjugated Organic Oligomers and Polymers. *J. Raman Spectrosc.* **1993**, *24* (8), 485–494.

(7) Schulz, H.; Baranska, M. Identification and Quantification of Valuable Plant Substances by IR and Raman Spectroscopy. *Vib. Spectrosc.* **2007**, *43* (1), 13–25.

(8) Heredia-Guerrero, J. A.; Benítez, J. J.; Domínguez, E.; Bayer, I. S.; Cingolani, R.; Athanassiou, A.; Heredia, A. Infrared and Raman Spectroscopic Features of Plant Cuticles: A Review. *Front. Plant Sci.* **2014**, *5*, 305.

(9) Prats Mateu, B.; Hauser, M. T.; Heredia, A.; Gierlinger, N. Waterproofing in Arabidopsis: Following Phenolics and Lipids In Situ by Confocal Raman Microscopy. *Front. Chem.* **2016**, *4* (10). https://doi.org/10.3389/fchem.2016.00010.

(10) Synytsya, A.; Čopíková, J.; Matějka, P.; Machovič, V. Fourier Transform Raman and Infrared Spectroscopy of Pectins. *Carbohydr. Polym.* **2003**, *54* (1), 97–106.

(11) Paczkowska, M.; Lewandowska, K.; Bednarski, W.; Mizera, M.; Podborska, A.; Krause, A.; Cielecka-Piontek, J. Application of Spectroscopic Methods for Identification (FT-IR, Raman Spectroscopy) and Determination (UV, EPR) of Quercetin-3-O-Rutinoside. Experimental and DFT Based Approach. *Spectrochim. Acta Part A Mol. Biomol. Spectrosc.* **2015**, *140*, 132–139.

(12) de Matos, Y. M. L. S.; Vasconcelos, D. L. M.; Barreto, A. C. H.; Rocha, J. E.; Neto, J. B.; Campina, F. F.; Tássia, T.; Sousa, A. K.; Teixeira, R. N. P.; Alvarez-Pizarro, J. C. Reduction of the Phytotoxic Effect of Mercury Chloride by Rutin and Evaluation of Interactions by Vibrational Spectroscopy (Raman and FTIR). *Vib. Spectrosc.* **2020**, *109*, 103084.

(13) Pompeu, D. R.; Larondelle, Y.; Rogez, H.; Abbas, O.; Pierna, J. A. F.; Baeten, V. Characterization and Discrimination of Phenolic Compounds Using Fourier Transform Raman Spectroscopy and Chemometric Tools. *BASE* **2018.**

(14) Zhou, C.; Diers, J. R.; Bocian, D. F. Q Y-Excitation Resonance Raman Spectra of Chlorophyll a and Related Complexes. Normal Mode Characteristics of the Low-Frequency Vibrations. *J. Phys. Chem. B* **1997**, *101* (46), 9635–9644.

(15) Schulz, H.; Baranska, M.; Baranski, R. Potential of NIR-FT-Raman Spectroscopy in Natural Carotenoid Analysis. *Biopolym. Orig. Res. Biomol.* **2005**, *77* (4), 212–221.

(16) Pascal, A.; Peterman, E.; Gradinaru, C.; van Amerongen, H.; van Grondelle, R.; Robert, B. Structure and Interactions of the Chlorophyll a Molecules in the Higher Plant Lhcb4 Antenna Protein. *J. Phys. Chem. B* **2000**, *104* (39), 9317–9321. https://doi.org/10.1021/jp001504m.

(17) Pascal, A.; Wacker, U.; Irrgang, K.-D.; Horton, P.; Renger, G.; Robert, B. Pigment Binding Site Properties of Two

Photosystem II Antenna Proteins: A Resonance Raman Investigation. *J. Biol. Chem.* **2000**, *275* (29), 22031–22036.

(18) Olejar, K. J.; Ricci, A.; Swift, S.; Zujovic, Z.; Gordon, K. C.; Fedrizzi, B.; Versari, A.; Kilmartin, P. A. Characterization of an Antioxidant and Antimicrobial Extract from Cool Climate, White Grape Marc. *Antioxidants* **2019**, *8* (7), 232.

(19) Merlin, J. C.; Cornard, J. P.; Stastoua, A.; Saidi-Idrissi, M.; Lautie, M. F.; Brouillard, R. Vibrational Analysis of Hydroxyflavylium Derivatives by IR, Fourier Transform Raman and Resonance Raman Spectroscopies. *Spectrochim. Acta Part A Mol. Spectrosc.* **1994**, *50* (4), 703–712.

(20) Conti, C.; Casati, M.; Colombo, C.; Possenti, E.; Realini, M.; Gatta, G. D.; Merlini, M.; Brambilla, L.; Zerbi, G. Synthesis of Calcium Oxalate Trihydrate: New Data by Vibrational Spectroscopy and Synchrotron X-Ray Diffraction. *Spectrochim. Acta Part A Mol. Biomol. Spectrosc.* **2015**, *150*, 721–730.

(21) Frost, R. L. Raman Spectroscopy of Natural Oxalates. *Anal. Chim. Acta* **2004**, *517* (1–2), 207–214.
